# PCGEM1 promotes cell proliferation and migration in endometriosis by targeting miR-124-3p-mediated ANTXR2 expression

**DOI:** 10.1186/s12905-023-02250-1

**Published:** 2023-03-13

**Authors:** Yong Liu, Chengmao Xie, Ting Li, Chang Lu, Linyuan Fan, Zhan Zhang, Sha Peng, Na Lv, Dan Lu

**Affiliations:** Department of Gynecology, Beijing Obstetrics and Gynecology Hospital, Capital Medical University, Beijing Maternal and Child Health Care Hospital, 100026 Beijing, China

**Keywords:** PCGEM1, miR-124-3p, ANTXR2, Endometriosis, Cell proliferation, Cell migration

## Abstract

**Background:**

Endometriosis, a common gynaecological disease in women, affects 10% of women of childbearing age. Among infertile women, this proportion is as high as 30–50%. Despite the high prevalence of endometriosis, the pathogenesis of endometriosis is still unclear.

**Methods:**

In the present study, bioinformatics analysis and molecular and animal experiments were employed to explore the functions of PCGEM1 in the pathogenesis of endometriosis. We established an endometriosis rat model and isolated endometrial stromal cells (ESCs) and primary normal ESCs (NESCs). Bioinformatics analysis was adopted to study the roles of PCGEM1 in promoting the pathogenesis of endometriosis. Luciferase reporter assays and RNA pull-down assays were carried out to study the mechanism by which PCGEM1 regulates ANTXR2.

**Results:**

Our results indicated that PCGEM1 promoted the motility and proliferation of ectopic endometrial cells, and the underlying mechanism was due to the direct binding of PCGEM1 to miR-124-3p to modulate ANTXR2 expression.

**Conclusion:**

PCGEM1 can influence endometrial stromal cell proliferation and motility and may be a novel therapeutic target for endometriosis.

**Supplementary Information:**

The online version contains supplementary material available at 10.1186/s12905-023-02250-1.

## Background

Endometriosis is a common gynaecological disease in women. The disease is characterized by the presence of endometrial-like glands and stromal outside the uterus, and it affects approximately 6–10% of women of childbearing age [1]. A variety of clinical manifestations, such as infertility, pelvic pain, dysmenorrhea and even malignant complications, are caused by endometriosis. This disease undoubtedly affects the quality of life of patients [2–4]. Although this disease has multiple relatively severe symptoms and a relatively high prevalence, the pathogenesis of endometriosis is still unclear. Thus, the need to find a diagnostic biomarker for endometriosis so that its pathogenesis can be explored is particularly urgent.

Long noncoding RNAs (lncRNAs), a class of transcripts discovered in the human genome with a length of > 200 nt, have no protein-coding ability [5]. LncRNAs are involved in numerous cellular processes mainly by regulating gene expression, which may contribute to the growth, drug resistance, and the migration of various cancer cells [6, 7]. For example, LINC00261 promotes cell proliferation and metastasis in endometriosis via downregulation of the miR-132-3p/BCL2L11 axis [8]. Downregulation of lncRNA-H19 can suppress the growth and invasion of ectopic endometrial cells by regulating miR-124-3p and ITGB3 [9]. The lncRNA PCGEM1 has been found to be associated with the incidence of prostate cancer (PCa) and can promote the progression of PCa [10, 11]. Recent studies have revealed that PCGEM1 acts as an oncogene in many cancers, including prostate cancer [12, 13], ovarian cancer [14, 15], gastric cancer [16] and renal cancer [17]. Furthermore, PCGEM1 expression levels have been found to be higher in endometrial carcinoma tissues. PCGEM1 also enhances endometrial carcinoma cell proliferation and apoptosis by targeting STAT3 via downregulation of miR-129 [18]. However, despite promising results indicating its oncogenic activity in endometrial carcinoma tissues, whether PCGEM1 exhibits similar activity in endometriosis remains unknown.

Given the importance of PCGEM1 in promoting endometrial carcinoma cell proliferation, we sought to determine whether PCGEM1 plays an important role in endometriosis. In this study, we established an endometriosis rat model, isolated ESCs and primary NESCs and studied the potential role of PCGEM1 in endometriosis. The promoting effects of PCGEM1 on cell motility and proliferation were observed. The mechanisms by which PCGEM1 regulates ANTXR2 expression to exert its effects were also investigated. Our study indicated that PCGEM1 may be a novel therapeutic target for endometriosis.

## Materials and methods

In this study, bioinformatics analysis and molecular and animal experiments were employed to explore the functions of PCGEM1. The detailed methodology is described in the following sections.

### Animals

Six- to eight-week-old female nonpregnant Sprague‒Dawley (SD) rats were obtained from the Animal Centre of Nanjing University. All rats were fed and cared for according to International Animal Care and Use Committee guidelines (IACUC)-approved protocols of Beijing Obstetrics and Gynaecology Hospital, Capital Medical University (No. ACU19-748). The rats (n = 20) were housed in a temperature-controlled room on a 12-h light/dark cycle, and tap water along with standard chow was provided for feeding. All methods are reported in accordance with the ARRIVE guidelines (https://arriveguidelines.org) for the reporting of animal experiments.

### Endometriosis rat model

To establish the endometriosis rat model, we referred to previous research reports [19]. SD rats were first anaesthetized, and a small incision was made in the centre of the abdomen. The left uterine horn was removed and immediately placed into saline to carefully separate the endometrium from the muscle. Then, the endometrium was divided into two pieces, of approximately 5 mm on each side. The subcutaneous pocket was ideally established on both sides of the abdominal wall, the uterine segment was ideally placed in the left and right spaces, and the endometrium ideally faced the abdominal muscle. The rats were injected subcutaneously immediately after the first laparotomy with 30 µg/kg oestradiol benzoate, and it was necessary to reinject the rats 10 days later to promote the growth of the autologous grafts. Four weeks after the operation, the grafts appeared raised and bright red, with a good surface vasculature and internal fluid accumulation. Microscopic histological observation showed that the transplanted endometrium survived and regenerated along the surface of the abdominal wall and was slightly thinner than the eutopic endometrium, with obvious hyperplasia of the surface epithelium and almost complete disappearance of the intrinsic glands in the stroma. Rats that met these criteria were included in the study; otherwise, they were not retained. In comparison, the incision, suture materials and graft placement positions in the rats in the control group were the same as those in the rats in the experimental group.

### Isolation of primary endometrial stromal cells

For isolation of NESCs and ESCs, we referred to previous studies [20]. Briefly, we used Hank’s balanced salt solution containing 1% penicillin/streptomycin, collagenase (1 mg/ml, 15 U/mg), HEPES (25 mmol/ml), (0.1 mg/ml, 1500 U/mg) and 1% streptomycin/penicillin to mince and digest the endometrial tissues from rats in the control and endometriosis model groups in a shaking water bath at 37 °C for 1 h. Then, the dispersed cells were isolated via filtration through a 40-µm cell strainer. We inoculated NESCs/ESCs in a 75 cm^2^ Falcon tissue culture flask, supplemented them with heat-inactivated 10% FBS and antibiotics in DMEM/Ham’s F12 medium (1:1) and then stored them in a humid environment at 37 °C. Cultured NESCs and ESCs were used between passages 3 and 5.

### HE staining

Endometriotic tissue was obtained from rats after anaesthesia and was then sliced into 5 μm thick sections. The sections were incubated in haematoxylin solution for 3–5 min after dewaxing and hydration and then incubated in ethanol––hydrochloric acid for 15 s. Subsequently, PBS solution was used to clean the sections, and then the sections were placed in eosin solution for 2–3 min. Finally, staining was evaluated under an optical microscope (Olympus, Japan).

### Real-time PCR

First, total RNA was extracted by TRIzol reagent (Invitrogen), and then cDNA was obtained with a reverse transcription kit (Invitrogen). Expression levels were calculated by using the 2 ^−ΔΔCT^ method. The primer sequence information is as follows:

**Table:** 

Primer Name	Sequence
PCGEM1-F	5’-TGCCTCAGCCTCCCAAGTAAC-3’
PCGEM1-R	5’-GGCCAAAATAAAACCAAACAT-3’
miR-124-3p-F	5′-CGTAAGGCACGCGGTGAA-3’
miR-124-3p-R	5′-AGTGCAGGGTCCGAGGTATT-3′
ANTXR2-F	5’-GGGGATCGGTTTGATGTGG-3’
ANTXR2-R	5’-GTGGGTTTGGGTCGAGGTG-3’
U6-F	5′-CTCGCTTCGGCAGCACATATACTA-3’
U6-R	5′-ACGAATTTGCGTGTCATCCTTGCG-3’
GAPDH-F	5′-GAGAAGTATGACAACAGCCTC-3’
GAPDH-R	5′-ATGGACTGTGGTCATGAGTC-3’

### Western blotting

Cells were incubated with plasmids (empty vector, PCGEM1 vector, ANTXR2 vector or shANTXR2 vector), interference fragments (NC, si-ANTXR2 or si-PCGEM1) or different plasmid/interference fragment combinations for 48 h and washed once with ice-cold PBS, and proteins were then extracted with sample buffer. First, approximately 50 µg of protein was loaded in an SDS‒PAGE gel and then transferred to PVDF membrane. Subsequently, the PVDF membrane was incubated with the primary antibody at 4 °C overnight and then incubated in milk containing the secondary antibody. The antibodies used in this experiment were as follows: anti-ANTXR2 (Abcam), anti-YAP1 (Abcam), anti-COL5A2 (Abcam), anti-VEGFC (Abcam) and anti-GAPDH (Proteintech). The membranes were cut prior to incubation with antibodies after protein transfer from the same gel.

### CCK-8 assay

We utilized a CCK-8 assay to test the viability of NESCs/ESCs. Cells were first seeded in a 96-well plate, and then every day, we added 10 µL of CCK-8 working solution to each well before measuring the absorbance at 450 nm by a microplate reader (BioTek, Winooski, VT, USA).

### Clone formation assay

For the clone formation assay, NESCs/ESCs were seeded in a 6-well plate (500 cells/well) and incubated for 2 weeks. Then, we fixed these cells with 2 ml methanol for 30 min before staining with crystal violet solution.

### Transwell invasion assay

We carried out the Transwell invasion assay with chambers containing membranes with 8.0-mm pores (Millipore). We first used 200 mL of foetal bovine serum-free DMEM to resuspend NESCs/ESCs and then plated all the above mentioned cells into the top compartments, which contained a Matrigel-coated membrane. After that, we added 500 ml medium containing 20% FBS into the bottom compartment as a chemoattractant for the cells. Forty-eight hours later, we fixed the invaded cells for 30 min before staining them with crystal violet solution. Finally, we used an inverted microscope to photograph and count the stained cells.

### Tube formation assay

For the tube formation assay, we added 50 µl of Matrigel into each well of a 96-well plate. NESCs/ESCs were seeded onto the Matrigel at a density of 30,000 cells/well after polymerization of the Matrigel at 37 °C for 30 min. Next, we incubated the cells in a humidified incubator at 37 °C and 5% CO_2_ for 6 h. Finally, an IX71 inverted microscope was used to observe the formed tubes.

### Dual luciferase reporter assay

pGL3-ANTXR2 reporter vectors were constructed by GeneCopoeia (Guangzhou, China) and transfected into cells by using Lipofectamine 3000 reagent. Forty-eight hours after transfection, the assay was performed with the Promega E1960 Dual‐Luciferase Reporter System.

### Pull-down assay

The direct interaction of miR-124-3p with ANTXR2 was evaluated by an RNA pull-down assay. First, we used cell lysis buffer (25 mM Tris-HCl, 2.5 mM EDTA, 0.05% NP-40, 70 mM KCl; pH 7.5) to harvest cells. Diluted protease inhibitor cocktail and an RNase inhibitor were both be added to the lysis buffer. Next, the cell lysate was incubated with the biotinylated double-stranded sequence of miR-124-3p (8 nmol) for 30 min at 4 °C with shaking at 8 rpm. Streptavidin Mutein Matrix was added, and then we obtained the protein–RNA complexes after centrifugation for 30 s at 5000×g. Finally, the complexes were eluted with elution buffer containing 80 U/ml RNase inhibitor. Finally, the precipitated complexes were analysed using qRT‒PCR and western blotting.

### Statistical analysis

Statistical analysis was implemented by using SPSS 18.0 and GraphPad 6.0. All data are shown as the means ± SDs. Moreover, Student’s t test and one-way ANOVA were used to compare data from two groups or more than two groups, respectively, and differences were considered statistically significant when the p value was less than 0.05.

## Results

### The lncRNA PCGEM1 and ANTXR2 are overexpressed in endometriosis

**Fig. 1:**
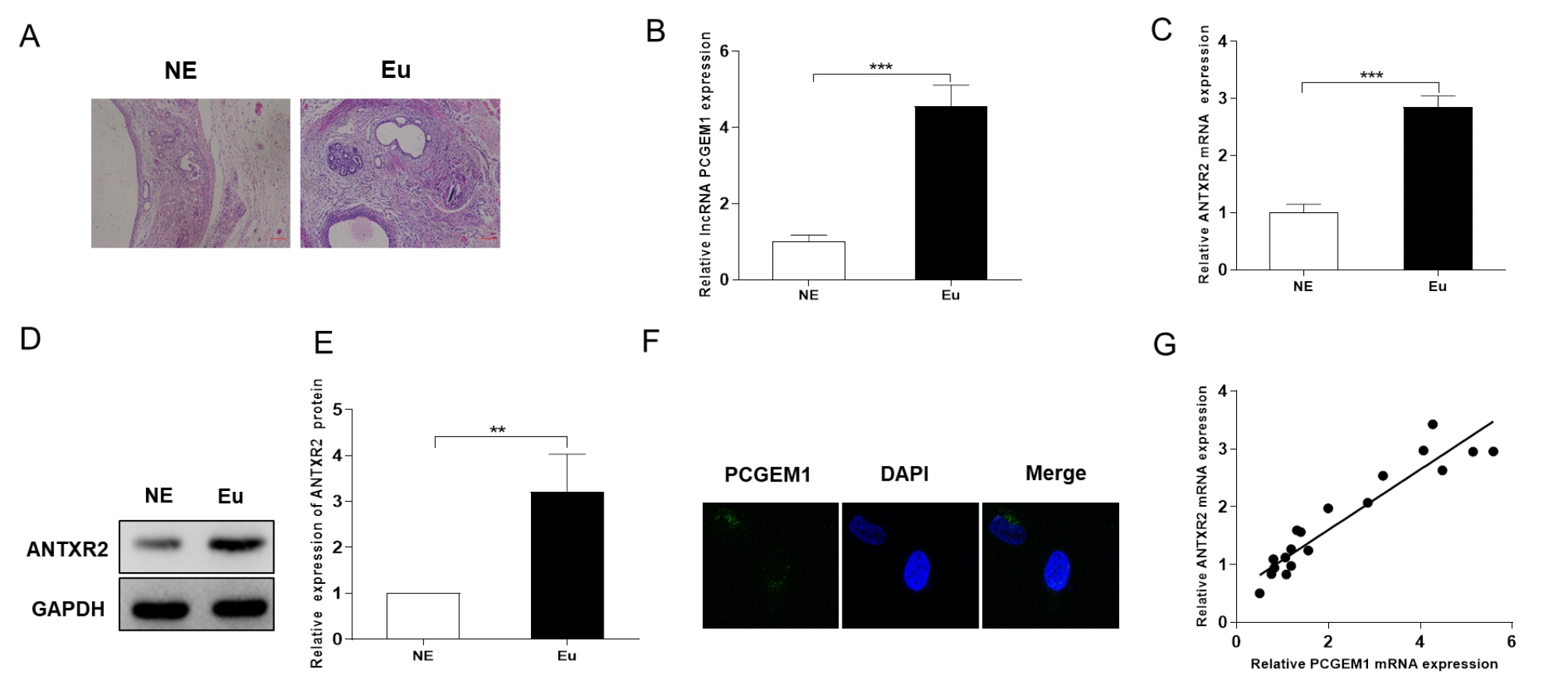
LncRNA PCGEM1 and ANTXR2 are overexpressed in endometriosis. (A) Representative H&E staining images of endometriotic and normal endometrial tissues. (B-C) Relative expression of PCGEM1 and ANTXR2 mRNA in different tissues (n = 10). (D-E) Relative expression of the ANTXR2 protein in different tissues (n = 3). The regions of the membrane corresponding to ANTXR2 and GAPDH were cut prior to incubation with antibodies after protein transfer from the same gel. (F) Intracellular localization of PCGEM1. (G) Pearson correlation analysis of PCGEM1 and ANTXR2 mRNA levels. (n = 10, R = 0.892, *P*<0.001). Scale bar = 20 μm. The values are presented as the means ± SDs. *P < 0.05, **P < 0.01, ***P < 0.001

To determine the potential role of lncRNA PCGEM1 in endometriosis, we established an endometriosis model in SD rats. H&E staining was used to confirm the lesions in the model (Fig. 1A). To interpret the biological functionality of the lncRNA PCGEM1 and anthrax toxin receptor 2 (ANTXR2) in endometriosis, we measured the PCGEM1 and ANTXR2 relative mRNA and protein expression levels in 10 paired ectopic (NE) and eutopic (Eu) endometrial tissues by qRT‒PCR (Fig. 1B-C) and Western blot analysis (Fig. 1D-E). As shown in Fig. 1B-C, the PCGEM1 and ANTXR2 mRNA expression levels were notably higher in the Eu group than in the NE group. Consistent with this result, the trend in the protein abundance was the same as the trend in mRNA expression (Fig. 1D-E). Moreover, PCGEM1 was localized in the cytoplasm, as shown in Fig. 1F. ANTXR2 has been reported to ameliorate endometriosis progression, and there is a significant increase in ANTXR2 mRNA and protein expression in endometriotic specimens [21]. Correlation analysis indicated that PCGEM1 mRNA expression was strongly correlated with ANTXR2 mRNA expression (r = 0.892, *P* < 0.001) (Fig. 1G). Taken together, these findings indicate that the lncRNA PCGEM1 and ANTXR2 are overexpressed in endometriosis.

### The lncRNA PCGEM1 promotes endometrial stromal cell proliferation by activating the ANTXR2 pathway

**Fig. 2:**
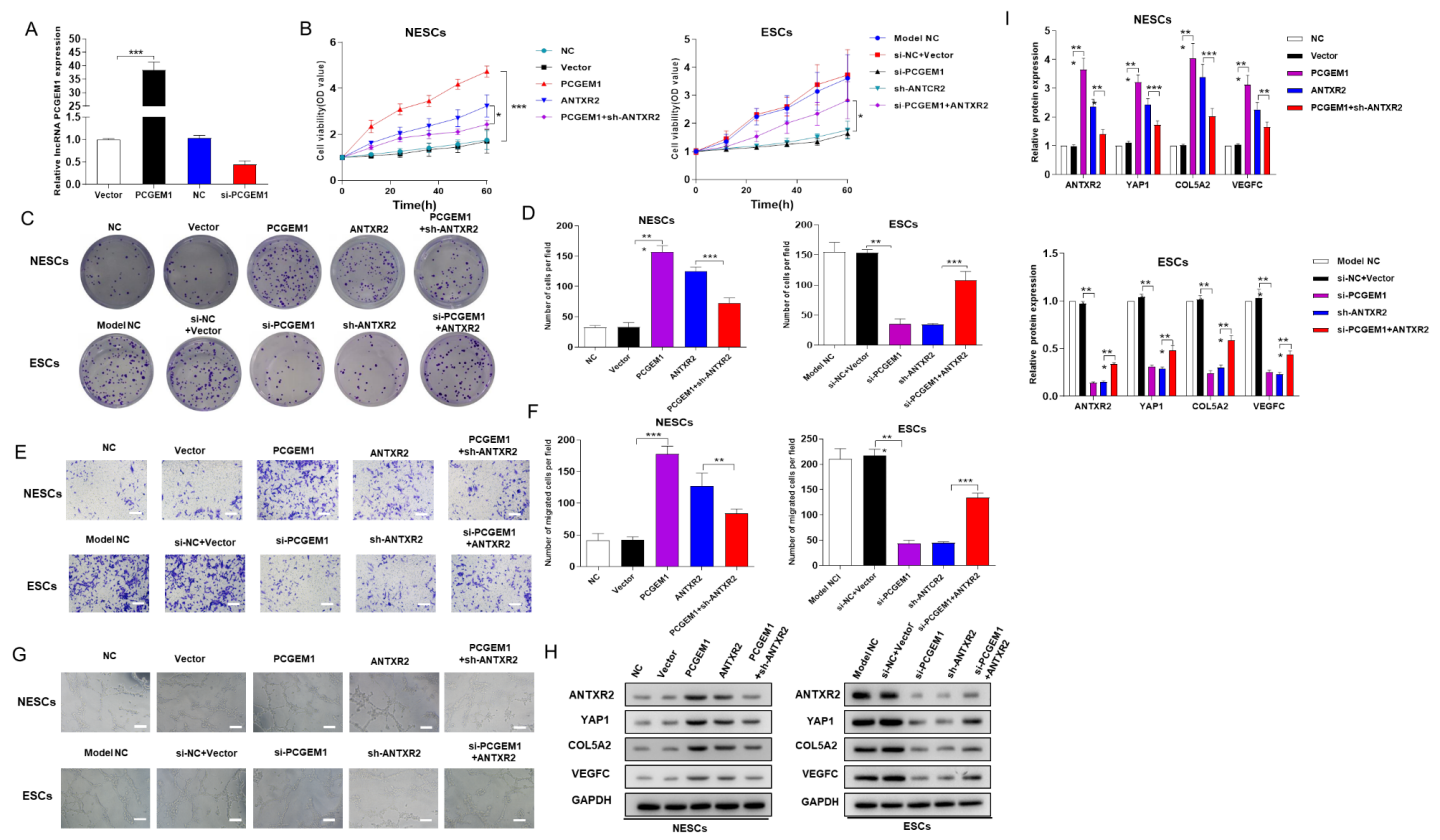
PCGEM1 stimulates endometrial stromal cell proliferation by activating the ANTXR2 pathway. (A) qPCR analysis of PCGEM1 expression in endometrial stromal cells (ESCs) after transfection with si-PCGEM1 for 24 h (n = 3). (B-D) Clone formation and CCK-8 assays were performed to assess normal endometrial stromal cell (NESC) and ESC proliferation after transfection (n = 3). (E-F) A Transwell assay was performed to evaluate the migration capacity of NESCs and ESCs (400× magnification) (n = 3). (G) A tube formation assay was carried out to evaluate the tube formation ability of NESCs and ESCs (n = 3). (H-I) Relative protein expression levels of ANTXR2 and its downstream proteins (including YAP1, COL5A2 and VEGFC) in NESCs and ESCs (n = 3). The membranes were cut prior to incubation with antibodies after protein transfer from the same gel. Scale bar = 20 μm. Mean ± SD from three independent tests. *P < 0.05, **P < 0.01, ***P < 0.001

NESCs and ESCs were obtained from normal and model SD rats, respectively, by enzymatic digestion. To further explore whether the effect of PCGEM1 on endometriosis is related to the overexpression of PCGEM1, PCGEM1-siRNA was adopted to ablate endogenous PCGEM1. After plasmid transfection, lncRNA PCGEM1 expression was downregulated in the PCGEM1 inhibitor group (Fig. 2A). To evaluate the possible role of PCGEM1 in ESCs, we quantified cell viability and migration using CCK8, clone formation and Transwell assays. In NESCs, overexpression of both PCGEM1 and ANTXR2 exhibited a notable increase compared with that in the normal control and vector-transfected cells (Fig. 2B-D). However, there was a significant decrease in the PCGEM1 + si-ANTXR2 group compared with the ANTXR2 group. In ESCs, si-PCGEM1 and si-ANTXR2 greatly reduced cell viability, and this decrease was alleviated when ANTXR2 expression was restored (Fig. 2B-D). Similar to their effects on cell viability, si-PCGEM1 and si-ANTXR2 inhibited cell migration, and this inhibition was mitigated when ANTXR2 expression was restored (Fig. 2E-F). Angiogenesis is a prerequisite for endometriotic lesion formation and development [19]. Thus, a tube formation assay was carried out to observe tube formation. Overexpression of PCGEM1 and ANTXR2 in NESCs greatly promoted tube formation, and tube formation was inhibited by cotransfection with PCGEM1 + sh-ANTXR2 (Fig. 2G). To further explore the expression of ANTXR2 and its downstream proteins (including YAP1, COL5A2 and VEGFC) in NESCs/ESCs, we measured the corresponding protein levels via Western blotting (Fig. 2H-I). Compared with that in control cells, PCGEM1 and ANTXR2 overexpression markedly increased the expression of the tested proteins (including ANTXR2, YAP1, COL5A2 and VEGFC) in NESCs, whereas downregulation of PCGEM1 and ANTXR2 decreased the relative expression of the proteins mentioned above (Fig. 2H-I). In summary, the results indicated that PCGEM1 stimulates the proliferation of ESCs by activating the ANTXR2 pathway.

### Identification of ANTXR2 as a new target of miR-124-3p

**Fig. 3:**
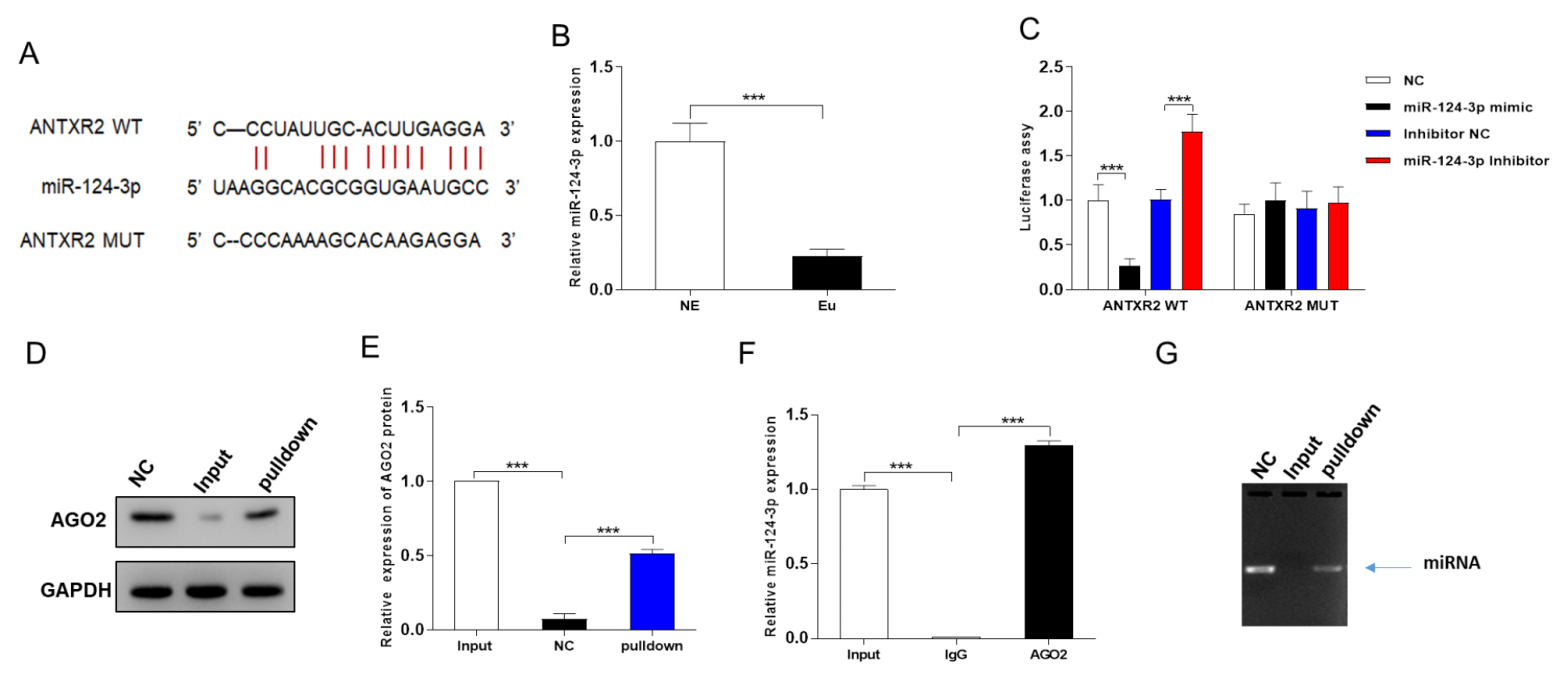
ANTXR2 is a target of miR-124-3p. (A) Prediction of the miR-124-3p binding sequence in the 3’UTR of ANTXR2. (B) Relative expression of miR-124-3p mRNA in different tissues (n = 3). (C) A dual luciferase reporter assay was used to investigate the relationship between miR-124-3p and ANTXR2 (n = 3). (D-E) An RNA pull-down assay was performed to evaluate the interaction between ANTXR2 and miR-124-3p (n = 3). The regions of the membrane corresponding to AGO2 and GAPDH were cut prior to incubation with antibodies after protein transfer from the same gel. (F-G) qPCR and immunoblotting analyses were adopted to verify the relationship between ANTXR2 and miR-124-3p expression (n = 3). Mean ± SD from three independent tests. *P < 0.05, **P < 0.01, ***P < 0.001

As shown in Fig. 3A, bioinformatics analysis suggested that there is a conserved binding sequence for miR-124-3p in the 3′UTR of ANTXR2, and we predicted that ANTXR2 was a target gene regulated by miR-124-3p. We then measured the mRNA levels of miR-124-3p both in NE and in Eu tissues by qRT‒PCR (Fig. 3B). As shown in Fig. 3B, the expression level of miR-124-3p in NE tissues was significantly different from that in Eu tissues. To further confirm that ANTXR2 is the target gene regulated by miR-124-3p, luciferase reporter and pull-down assays were adopted to test the regulatory effect of miR-124-3p on ANTXR2 (Fig. 3C-G). The results showed that miR-124-3p mimic transfection significantly inhibited the activity of the reporter gene, while the luciferase activity of ANTXR2-WT was significantly increased in the miR-124-3p inhibitor group, and both of these effects were abolished after the putative binding site was mutated (Fig. 3C). Similarly, the pull-down assay results confirmed the results of the luciferase reporter assay (Fig. 3D-G). Collectively, these results indicated that ANTXR2 is directly regulated by miR-124-3p.

### PCGEM1 regulates ANTXR2 expression in ectopic stromal cells by suppressing mir-124-3p expression

**Fig. 4:**
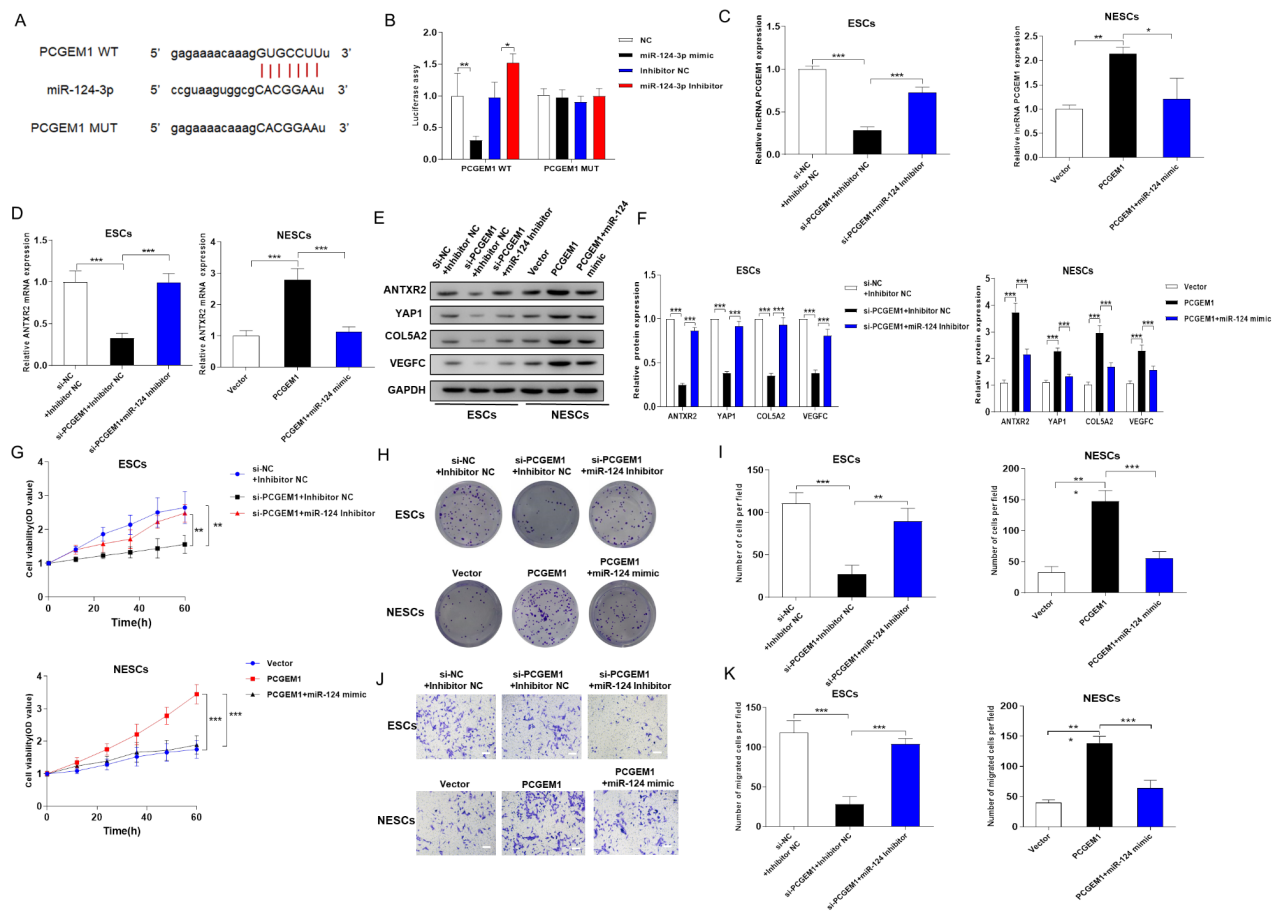
PCGEM1 promotes ANTXR2 expression in ectopic stromal cells by inhibiting miR-124-3p expression. (A) Prediction of the miR-124-3p binding sequence in the 3’UTR of PCGEM1(n = 3). (B) A dual luciferase reporter assay was performed to investigate the relationship between PCGEM1 and miR-124-3p (n = 3). (C-D) Relative expression of PCGEM1 and ANTXR2 mRNA in different cells (n = 3). (E-F) Relative expression of ANTXR2 and its downstream proteins (including YAP1, COL5A2 and VEGFC) in NESCs and ESCs (n = 3). The membranes were cut prior to incubation with antibodies after protein transfer from the same gel. (G-I) Clone formation and CCK-8 assays were performed to assess NESC and ESC proliferation after transfection. (J-K), The motility of NESCs/ESCs (400× magnification). Scale bar = 20 μm. Mean ± SD from three independent tests. *P < 0.05, **P < 0.01, ***P < 0.001

Considering that ANTXR2 is a target of miR-124-3p, we then predicted that miR-124-3p is a potential miRNA associated with the function of PCGEM1 through starBase (Fig. 4A). Next, a luciferase reporter assay was carried out to confirm the interaction between PCGEM1 and miR-124-3p. The results showed that miR-124-3p mimic transfection significantly inhibited the activity of the reporter gene, while miR-124-3p inhibitor transfection significantly increased the luciferase activity of ANTXR2-WT, and both of these effects were abolished after the putative binding sites were mutated (Fig. 4B). To further identify whether PCGEM1 performs its function in ESCs via the miR-124-3p/ANTXR2 axis, we conducted rescue experiments. First, the RT‒qPCR results verified the transfection efficiency of si-PCGEM1 + the miR-124-3p inhibitor and PCGEM1 + the miR-124-3p mimic separately in NESCs/ESCs (Fig. 4C). Next, the relative mRNA expression of ANTXR2 was measured by qRT‒PCR, which indicated that ANTXR2 mRNA expression was inhibited by knockdown of PCGEM1 and was promoted by miR-124-3p inhibitor transfection in ESCs. Similarly, PCGEM1 promoted ANTXR2 mRNA expression, and then the baseline expression level was restored when the miR-124-3p mimic was transfected (Fig. 4D). Immunoblot analysis suggested that the expression of ANTXR2 and its downstream proteins, including YAP1, COL5A2 and VEGFC, was suppressed by knockdown of PCGEM1 and restored after miR-124-3p inhibitor transfection in ESCs (Fig. 4E-F). Correspondingly, all of the above protein levels were increased by PCGEM1 overexpression and inhibited by miR-124-3p mimic transfection (Fig. 4E-F). Moreover, the results of both the clone formation and CCK-8 assays revealed that ESC proliferation was suppressed by si-PCGEM1 transfection and was facilitated by miR-124-3p inhibitor transfection (Fig. 4G-I). NESC proliferation was promoted by PCGEM1 overexpression and inhibited by miR-2278 mimic transfection (Fig. 4G-I). Furthermore, PCGEM1 overexpression accelerated cell migration, which was suppressed by si-PCGEM1 transfection and was facilitated by miR-124-3p inhibitor transfection (Fig. 4J-K). Overall, we concluded that PCGEM1 promotes ANTXR2 expression in ectopic stromal cells by inhibiting miR-124-3p expression.

## Discussion

Recently, great progress has been made in the treatment and diagnosis of endometriosis, but there are ways to further explore effective therapies for this disease. The complexity of healing endometriosis stems from our lack of understanding of the heterogeneity and pathophysiological mechanisms of the disease [3, 4]. The lncRNA PCGEM1 is a relatively novel lncRNA, and a previous study demonstrated that PCGEM1 is capable of enhancing endometrial carcinoma cell proliferation and apoptosis by targeting STAT3 via sponging of miR-129 [18]. Convincing evidence has proven that differences in the subcellular localization determine the distinct biological functions of lncRNAs [22]. Nuclear lncRNAs can directly regulate transcription factors [23], while cytoplasmic lncRNAs can sponge mRNAs or miRNAs [24]. In our study, PCGEM1 was found to be localized in the cytoplasm and stimulated ESC cell growth and migration by regulating the miR-124-3p/ANTXR2 axis.

An accumulating body of evidence indicates that miR-124-3p dysregulation is likely to be one of the key factors in tumorigenesis in diverse cancers, such as bladder cancer [25], gastric cancer [26], breast cancer [27], hepatocellular carcinoma [28], endometrial cancer [29] and cervical cancer [30]. Our results indicated that miR-124-3p is downregulated in endometriotic tissue samples, and encouragingly, the reciprocal repression between PCGEM1 and miR-124-3p was revealed in the present study.

ANTXR2 (also called CMG2), the second receptor for the anthrax toxin, is a transmembrane protein [31]. Most studies of ANTXR2 have focused on its role in the pathogenesis of anthrax [32]. Surprisingly, reports have shown that *Antxr2* knockout female mice are unable to deliver pups because of uterine dysfunction. Thus, this finding indicates that *ANTXR2* plays an essential role in female procreation [31]. Furthermore, ANTXR2 has been reported to ameliorate endometriosis progression, and both the mRNA and protein levels showed a significant increase in endometriotic specimens [21]. In our study, PCGEM1 was found to stimulate the proliferation/migration of endometrial stromal cells by activating the ANTXR2 pathway, and sequence analysis showed that PCGEM1 could bind to miR-124-3p. The results demonstrated that PCGEM1 could promote ANTXR2 expression in ectopic endometrial stromal cells by suppressing miR-124-3p expression.

In addition to the role of lncRNAs in the pathogenesis of endometriosis, accumulating evidence suggests that immune cells, adhesion molecules, extracellular matrix metalloproteinases and proinflammatory cytokines activate/alter the peritoneal microenvironment, creating conditions conducive to the differentiation, adhesion, proliferation and survival of ectopic endometrial cells [33–35]. In patients with endometriosis, the density of nerve fibres in the peritoneum is significantly altered, providing a new perspective for understanding the development of endometriosis and the treatment of endometriosis-related pain [34]. Furthermore, the changes in M1 macrophages and M2 macrophages from phase I to phase IV showed an opposite trend, which may be related to the pathogenesis of endometriosis [33]. Endometriotic tissue has been found to harbour cancer-related mutations and miRNA dysregulation associated with carcinogenesis, even if the tissue appears benign under microscopic examination, further demonstrating how endometriosis and cancer share some common pathways and highlighting new theories about the pathogenesis of endometriosis [35].

Due to the complexity of endometriosis, there is still no effective molecular marker to indicate its occurrence [36–38]. Although several theories have been proposed, the pathogenesis of endometriosis is still controversial: immune, environmental, endocrine and genetic factors are all thought to explain the complex mechanism underlying the origin and development of ectopic endometrium [36]. The molecular and cellular differences between eutopic and ectopic endometrium are more complex, especially regarding the expression of neurotrophic factors, which play a key role in the aggravation of pain [37, 38]. This suggests that a change in one or several factors may not be enough to indicate the occurrence of the disease, posing a new challenge in studying the pathogenesis of endometriosis.

The limitations of this research, including the lack of information about the cellular localization of PCGEM1, remain to be addressed and will direct research into the most fundamental mechanism. Second, the role of the PCGEM1/miR-124-3p/ANTXR2 axis needs to be verified in vivo. The connections with the clinical features of patients need to be verified in future studies with a large group of samples. Further research is still needed to completely define the function and mechanism of PCGEM1.

## Conclusion

We investigated the potential roles of PCGEM1 in the pathogenesis of endometriosis. PCGEM1 promotes cell proliferation and migration in endometriosis. Furthermore, we provided evidence that PCGEM1 regulates ANTXR2 expression to accelerate disease progression by binding to miR-124-3p directly. The PCGEM1/miR-124-3p/ANTXR2 regulatory network is likely to be a novel therapeutic target in endometriosis.

## Electronic supplementary material

Below is the link to the electronic supplementary material.

Additional File: Table of Contents：Western blot raw data

## Data Availability

All relevant data are provided within the paper. There is no analysis of data such as proteomics data and protein sequences, DNA and RNA sequences, genetic polymorphisms, linked genotype and phenotype data, macromolecular structures, gene expression data and crystallographic data for small molecules in the manuscript. The materials used in the study can be requested through the corresponding author.

## Abbreviations

LncRNA: Long noncoding RNA
ANTXR2: Anthrax toxin receptor 2
SD: Sprague‒Dawley
IACUC: International Animal Care and Use Committee
NESCs: normal endometrial stromal cells
ESCs: Endometrial stromal cells
PCGEM1: Prostate cancer gene expression marker 1.
