# Supplementary material for: PCGEM1 promotes cell proliferation and migration in endometriosis by targeting miR-124-3p-mediated ANTXR2 expression
Source: BMC Womens Health. 2023 Mar 13;23:104. doi: 10.1186/s12905-023-02250-1 (PMC10012497; doi:10.1186/s12905-023-02250-1)
Supplement: Supplementary file 1 — Additional File: Table of Contents：Western blot raw data [file 12905_2023_2250_MOESM1_ESM.pdf]

Supporting Information

PCGEM1 promotes cell proliferation and migration in endometriosis by targeting miR-124-3p-mediated ANTXR2 expression

Yong Liu[#], Chengmao Xie[#], Ting Li, Chang Lu, Linyuan Fan, Zhan Zhang, Sha Peng, Na Lv, Dan Lu*

All of the authors are from the same institution:

Department of Gynecology, Beijing Obstetrics and Gynecology Hospital, Capital Medical University. Beijing Maternal and Child Health Care Hospital, Beijing, 100026, China.

First author:

Yong Liu, E-mail: youngleu@ccmu.edu.cn. ORCID: 0000-0003-2030-643X.

co-first author:

Chengmao Xie, E-mail: xiechengmao@ccmu.edu.cn

#: These authors contributed equally to this work.

***Corresponding author:**

Dan Lu, Department of Gynecology, Beijing Obstetrics and Gynecology Hospital, Capital Medical University. Beijing Maternal and Child Health Care Hospital, Beijing, 100026, China. E-mail: ludan6268@ccmu.edu.cn. Tel: +86-010-52272515. ORCID: 0000-0001-6275-1158.

Table of Contents

Western blot raw data……………………………........................ 2-17

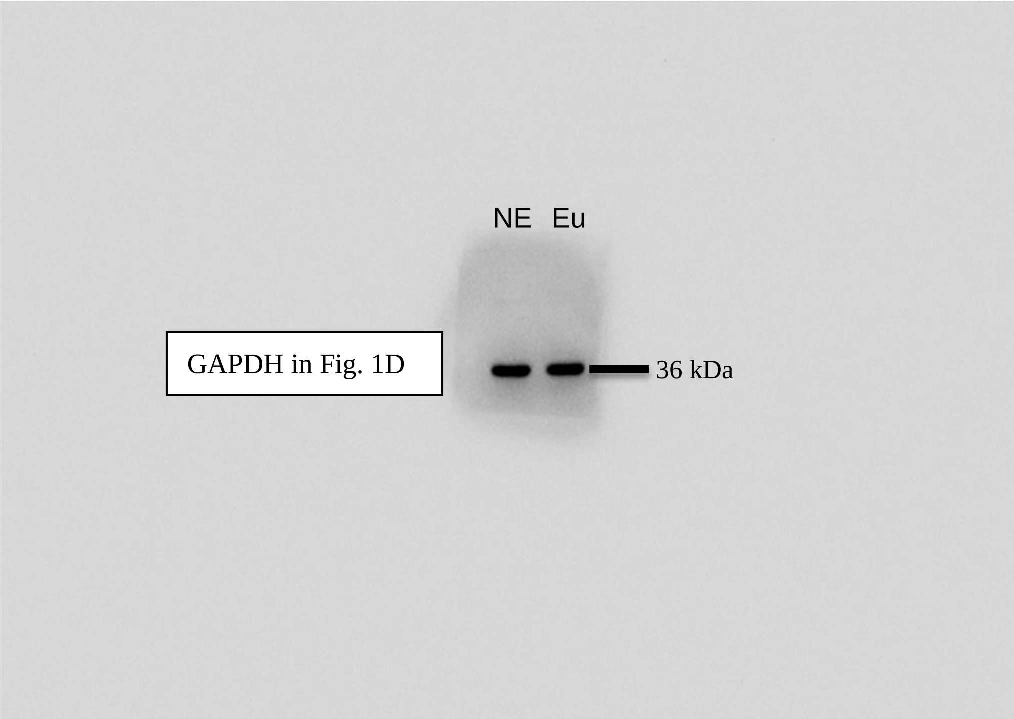
NE Eu
GAPDH in Fig. 1D
36 kDa

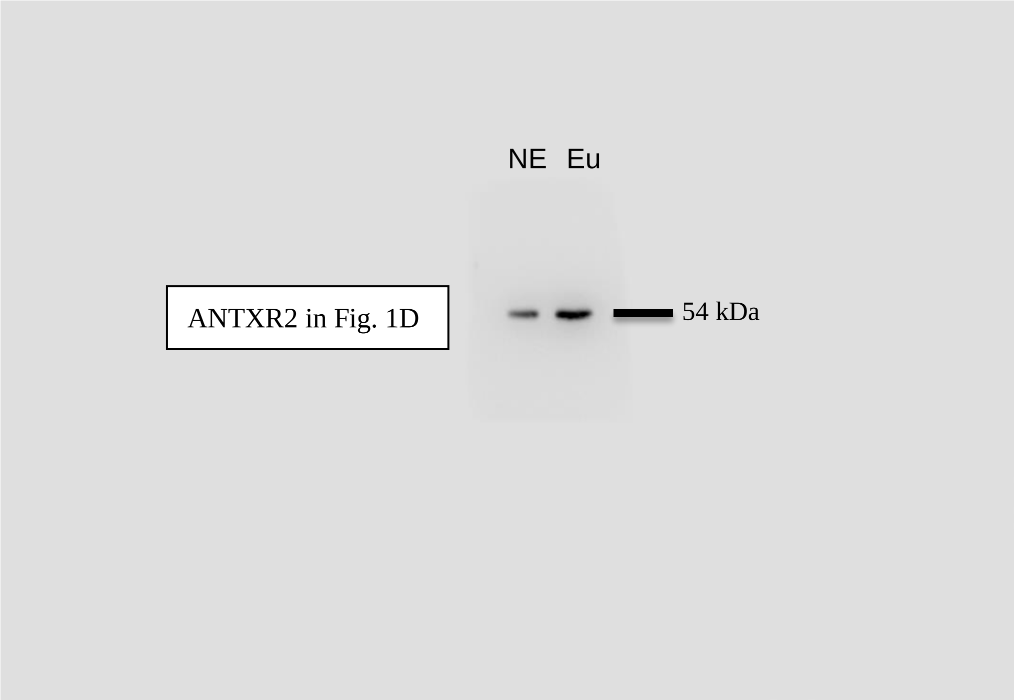
NE Eu
ANTXR2 in Fig. 1D
54 kDa

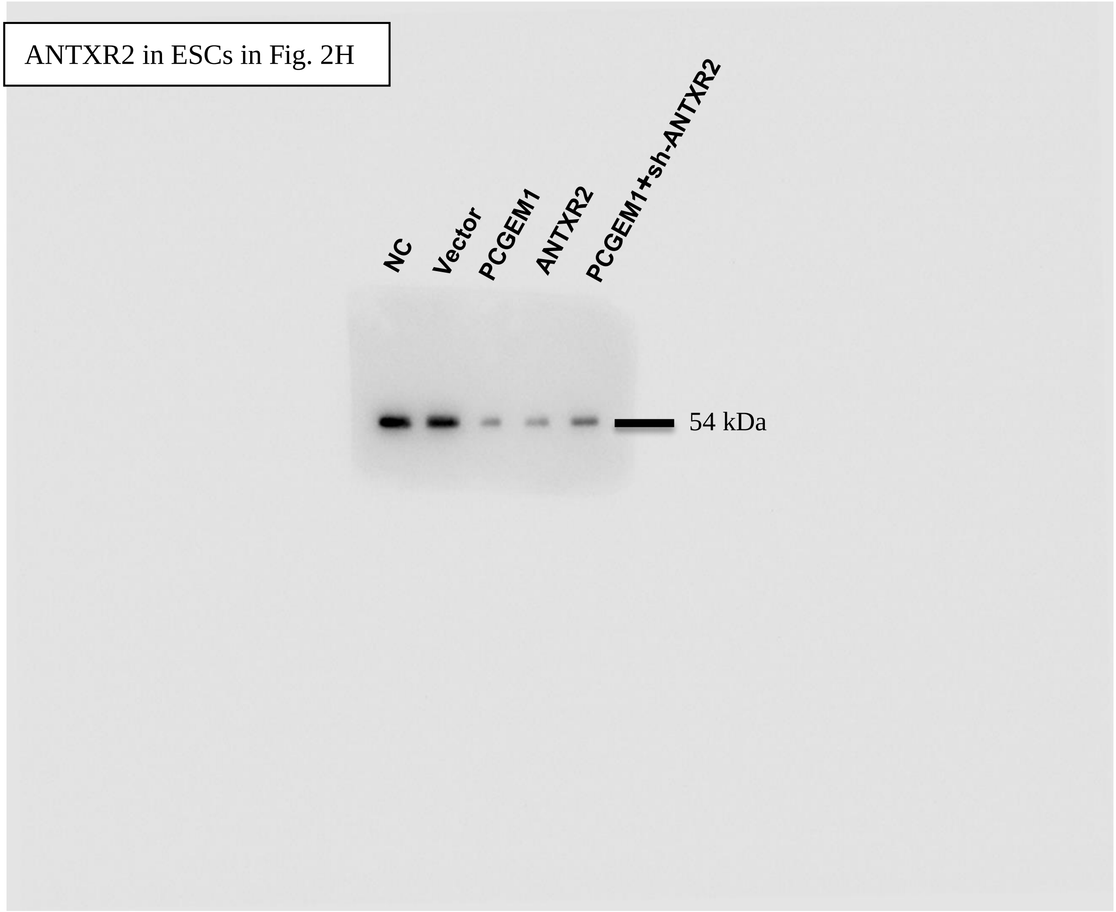
ANTXR2 in ESCs in Fig. 2H
NC
Vector
PCGEM1
ANTXR2
PCGEM1+sh-ANTXR2
54 kDa

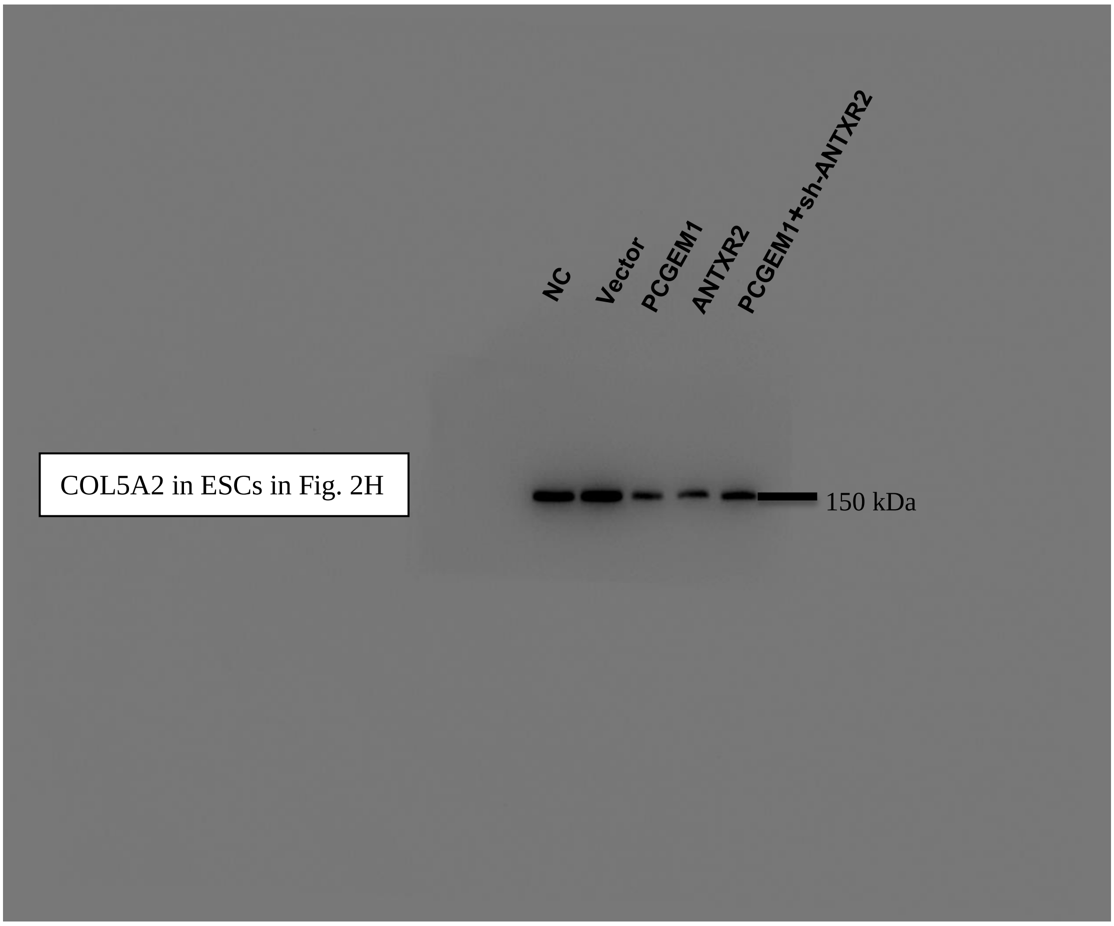
NC
Vector
PCGEM1
ANTXR2
PCGEM1+sh-ANTXR2
COL5A2 in ESCs in Fig. 2H
150 kDa

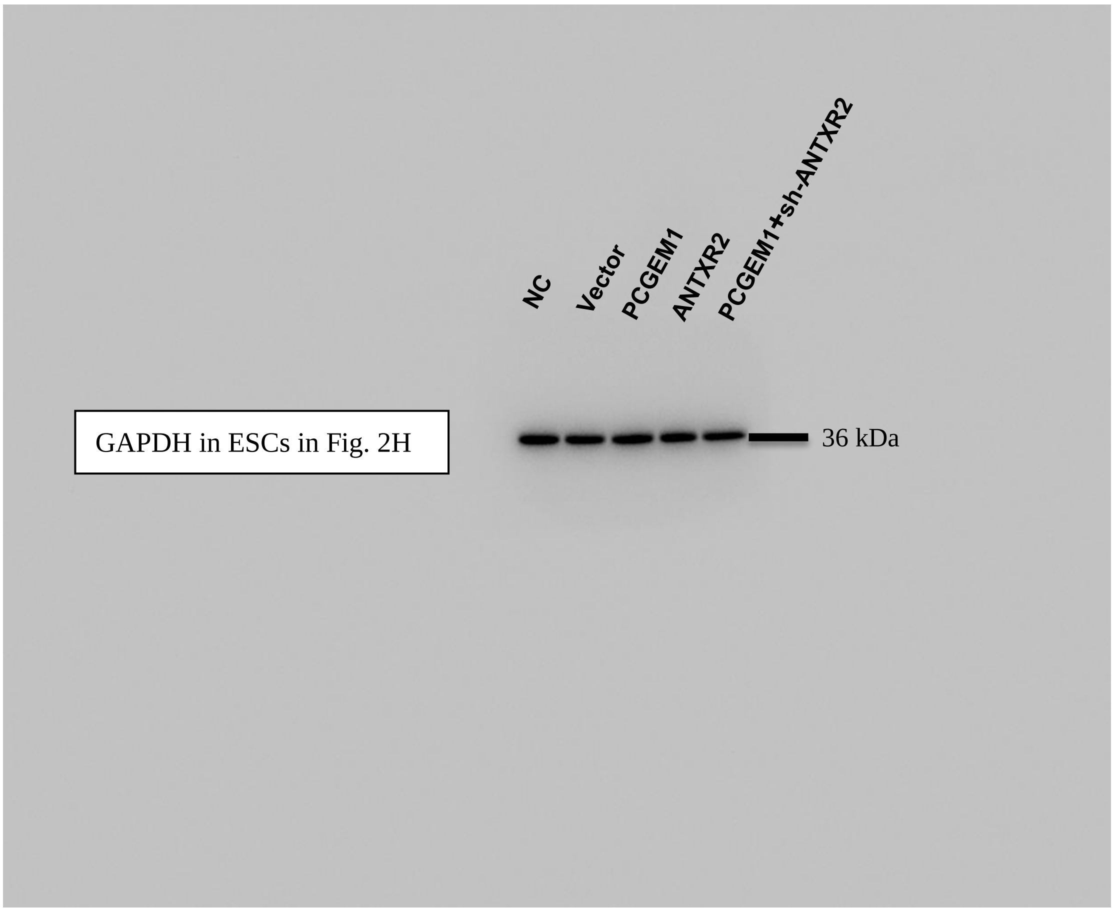
NC
Vector
PCGEM1
ANTXR2
PCGEM1+sh-ANTXR2
GAPDH in ESCs in Fig. 2H
36 kDa

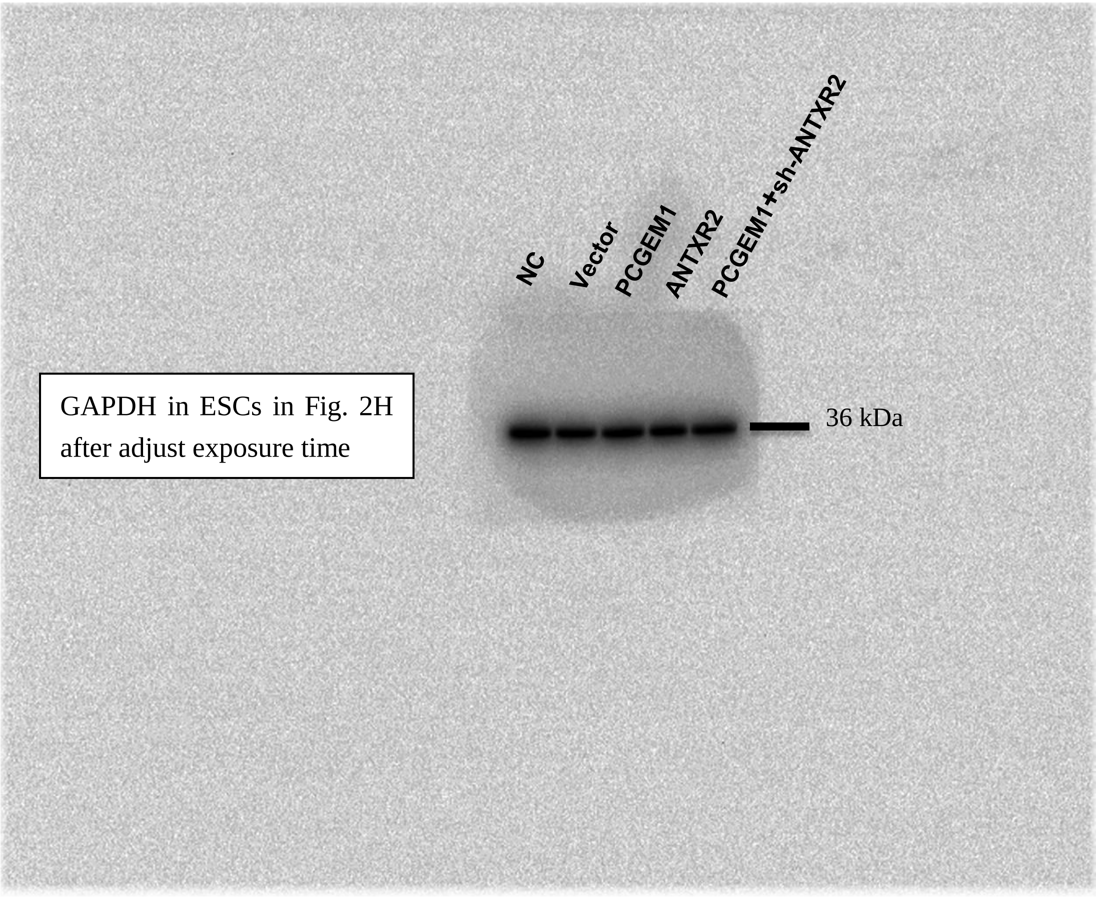
NC
Vector
PCGEM1
ANTXR2
PCGEM1+sh-ANTXR2
GAPDH in ESCs in Fig. 2H after adjust exposure time
36 kDa

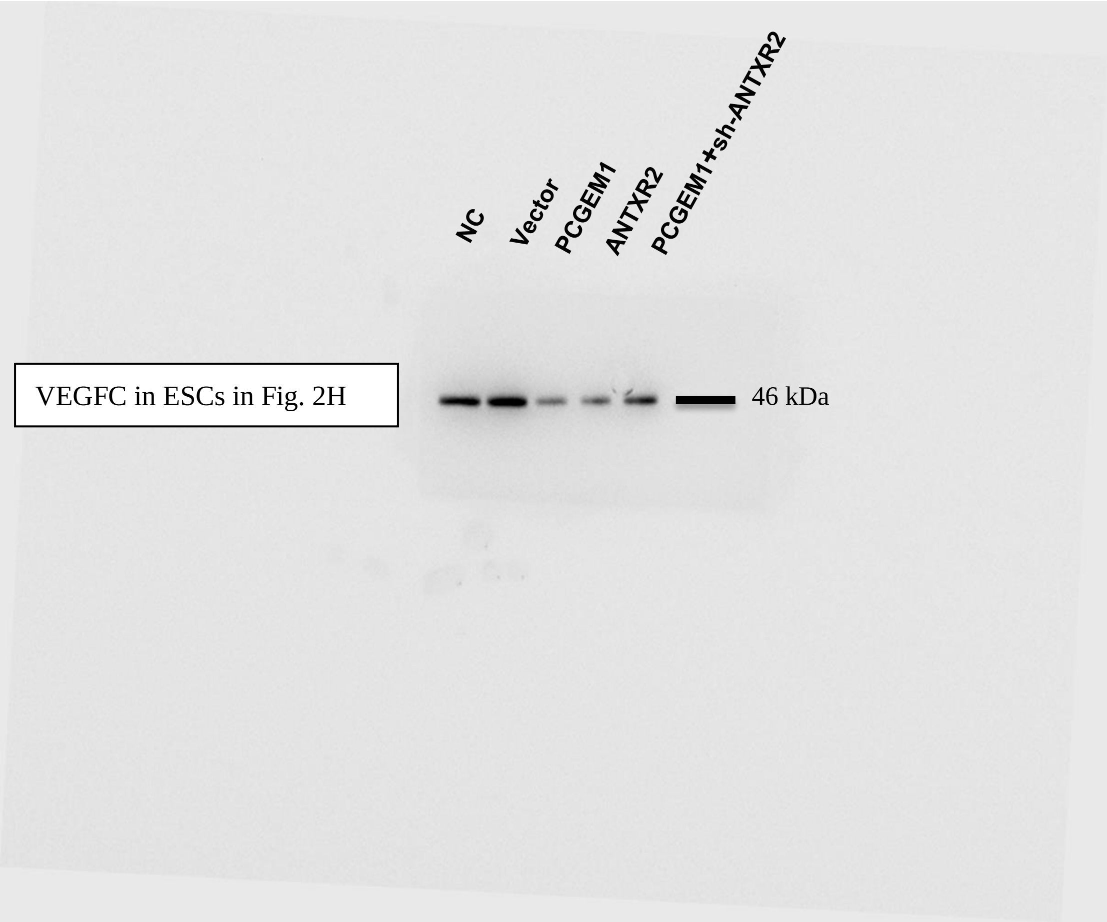
NC
Vector
PCGEM1
ANTXR2
PCGEM1+sh-ANTXR2
VEGFC in ESCs in Fig. 2H
46 kDa

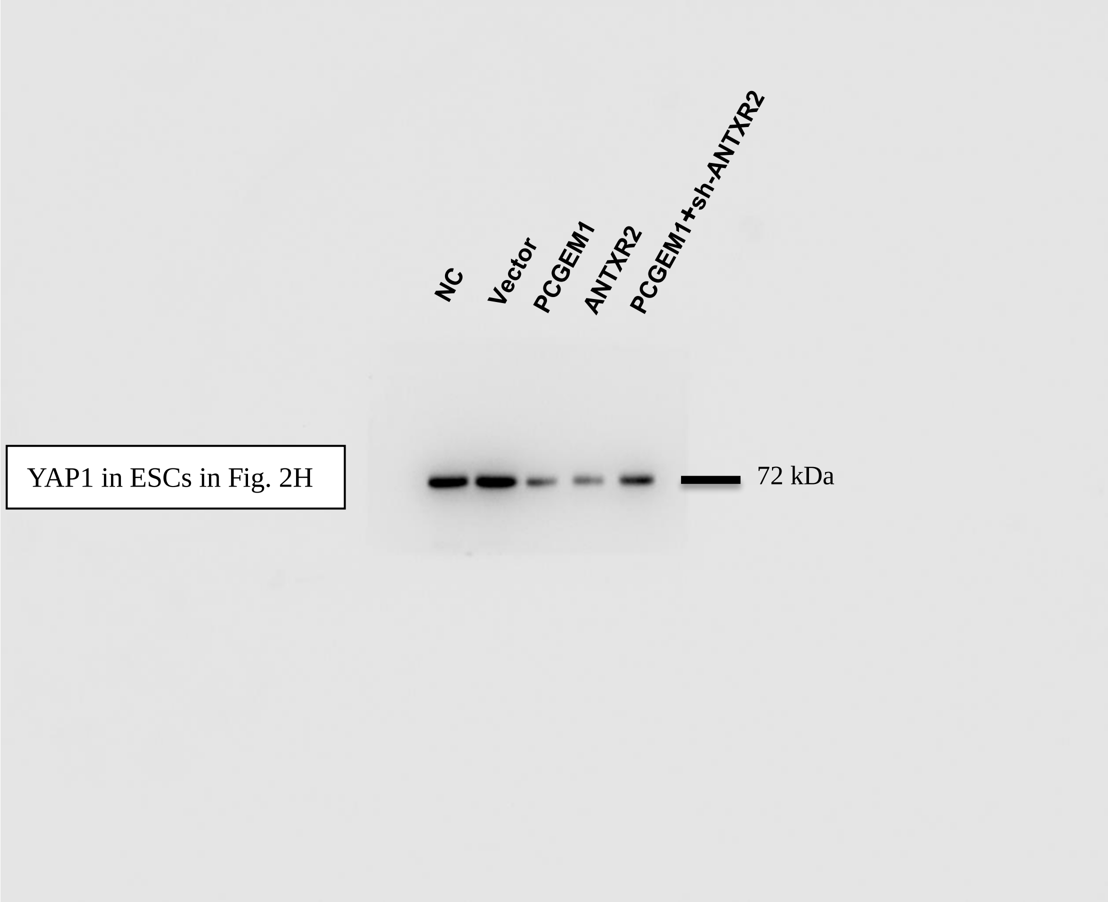
NC
Vector
PCGEM1
ANTXR2
PCGEM1+sh-ANTXR2
YAP1 in ESCs in Fig. 2H
72 kDa

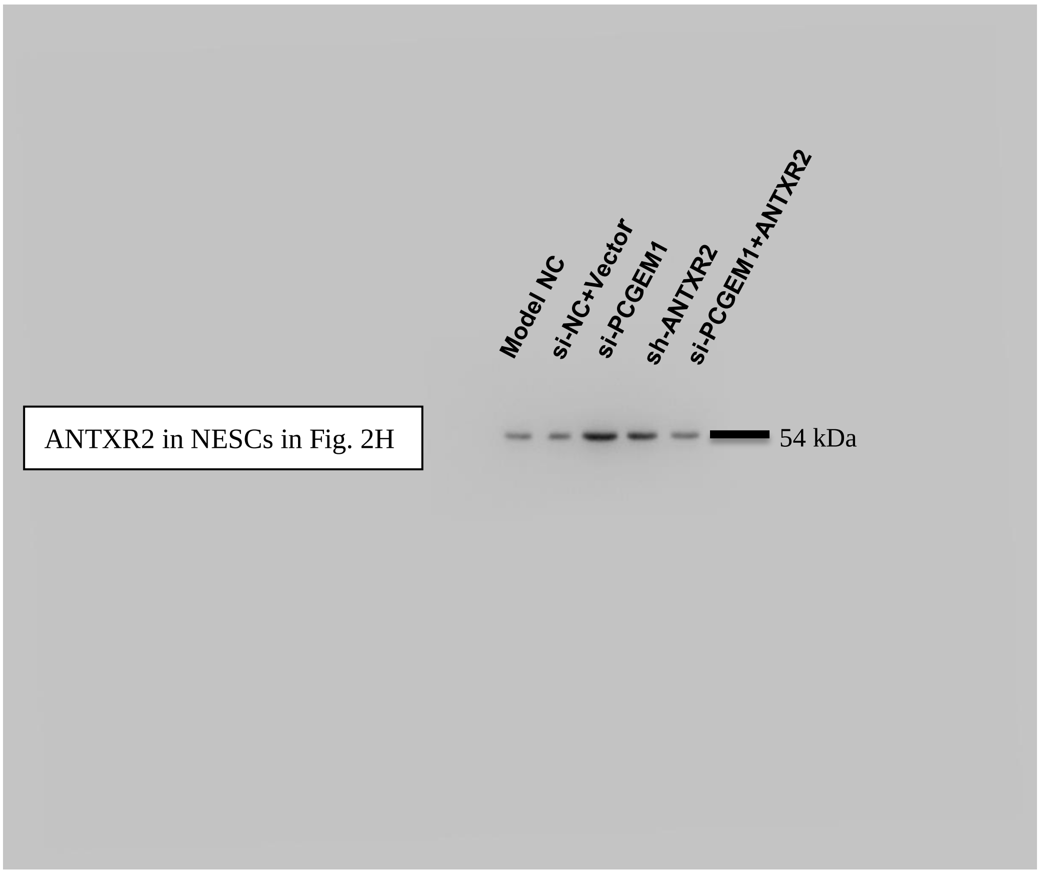
Model NC
si-NC+Vector
si-PCGEM1
sh-ANTXR2
si-PCGEM1+ANTXR2
ANTXR2 in NESCs in Fig. 2H
54 kDa

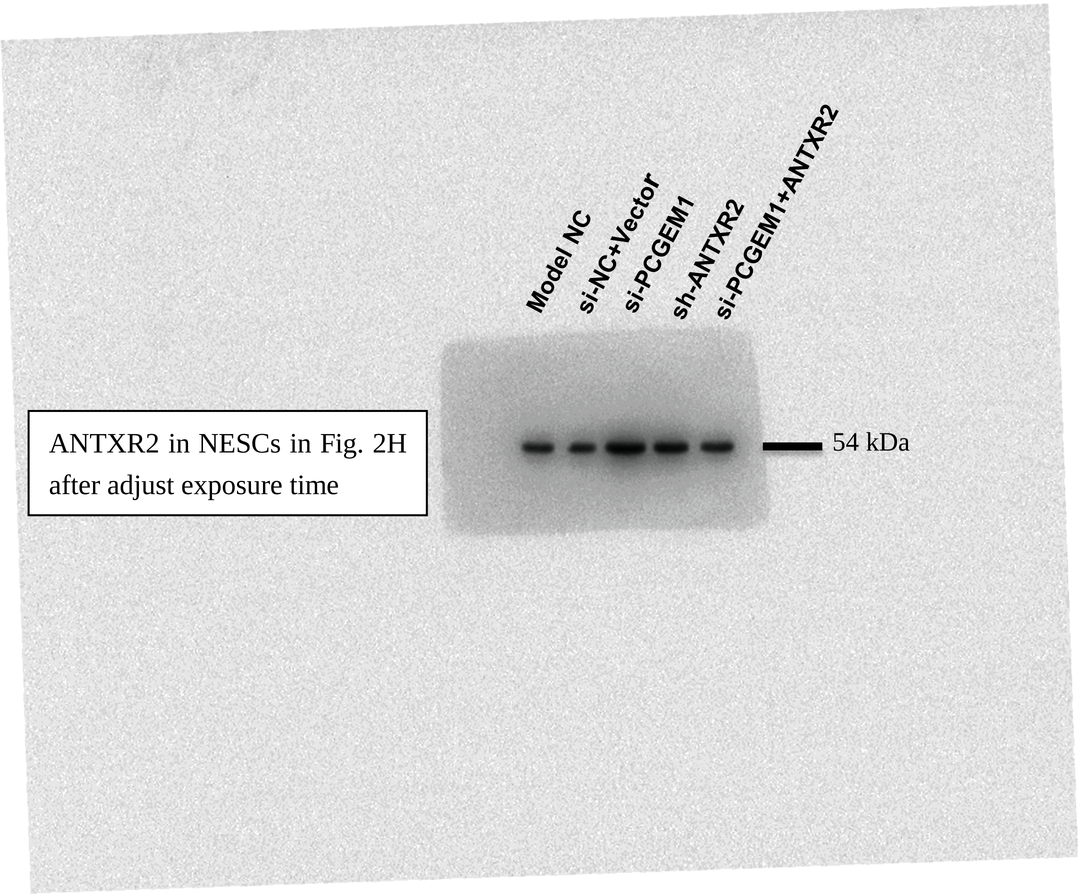
Model NC
si-NC+Vector
si-PCGEM1
sh-ANTXR2
si-PCGEM1+ANTXR2
ANTXR2 in NESCs in Fig. 2H
after adjust exposure time
54 kDa

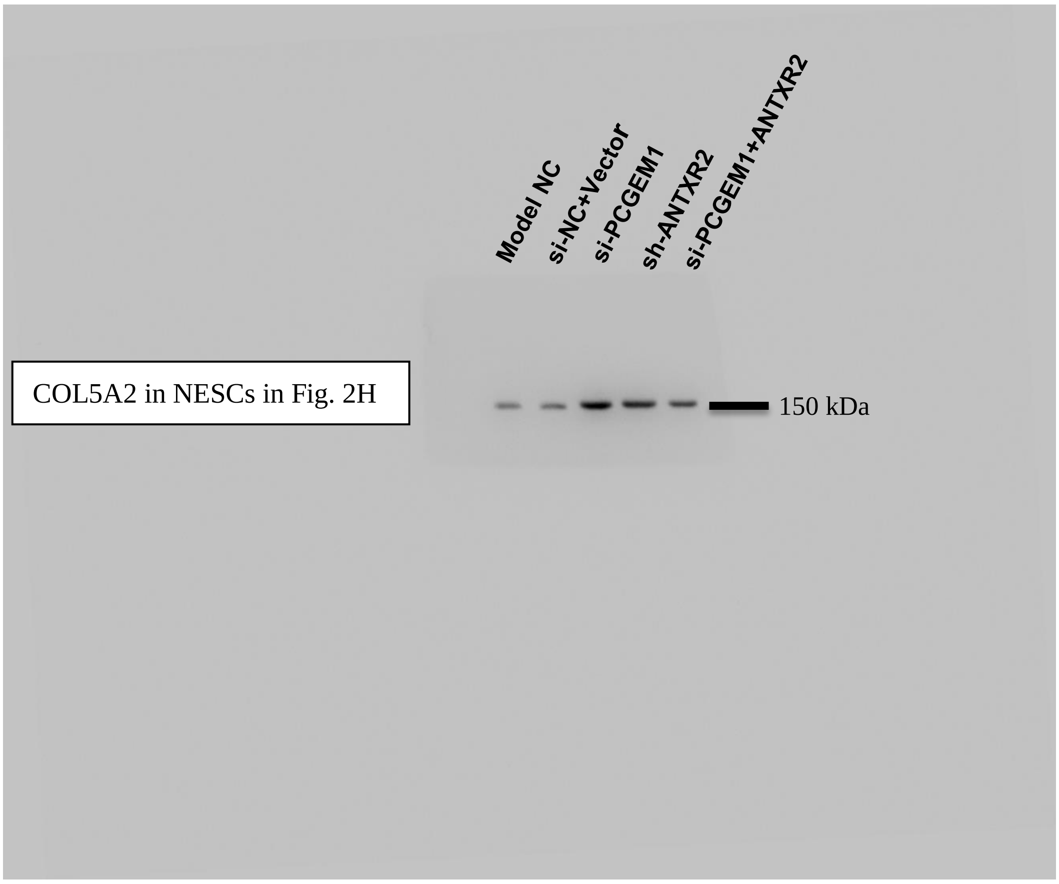

Model NC
si-NC+Vector
si-PCGEM1
sh-ANTXR2
si-PCGEM1+ANTXR2
COL5A2 in NESCs in Fig. 2H
150 kDa

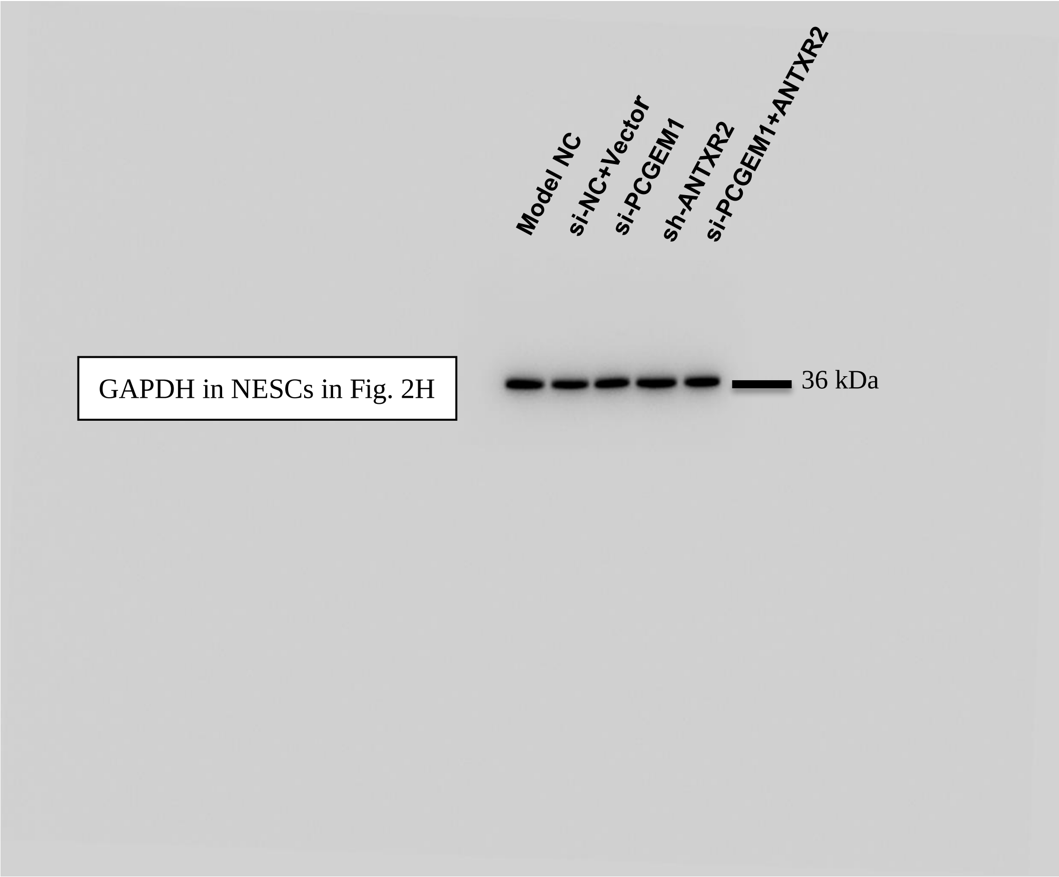
Model NC
si-NC+Vector
si-PCGEM1
sh-ANTXR2
si-PCGEM1+ANTXR2
GAPDH in NESCs in Fig. 2H
36 kDa

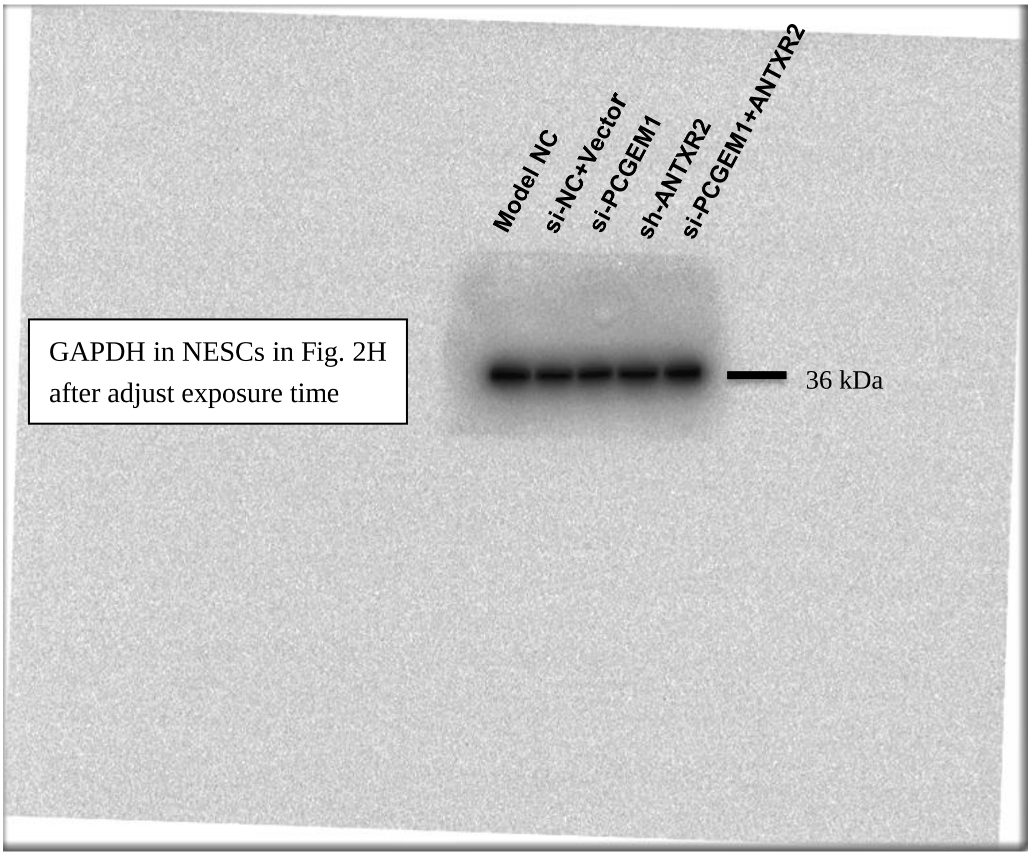
Model NC
si-NC+Vector
si-PCGEM1
sh-ANTXR2
si-PCGEM1+ANTXR2
GAPDH in NESCs in Fig. 2H
after adjust exposure time
36 kDa

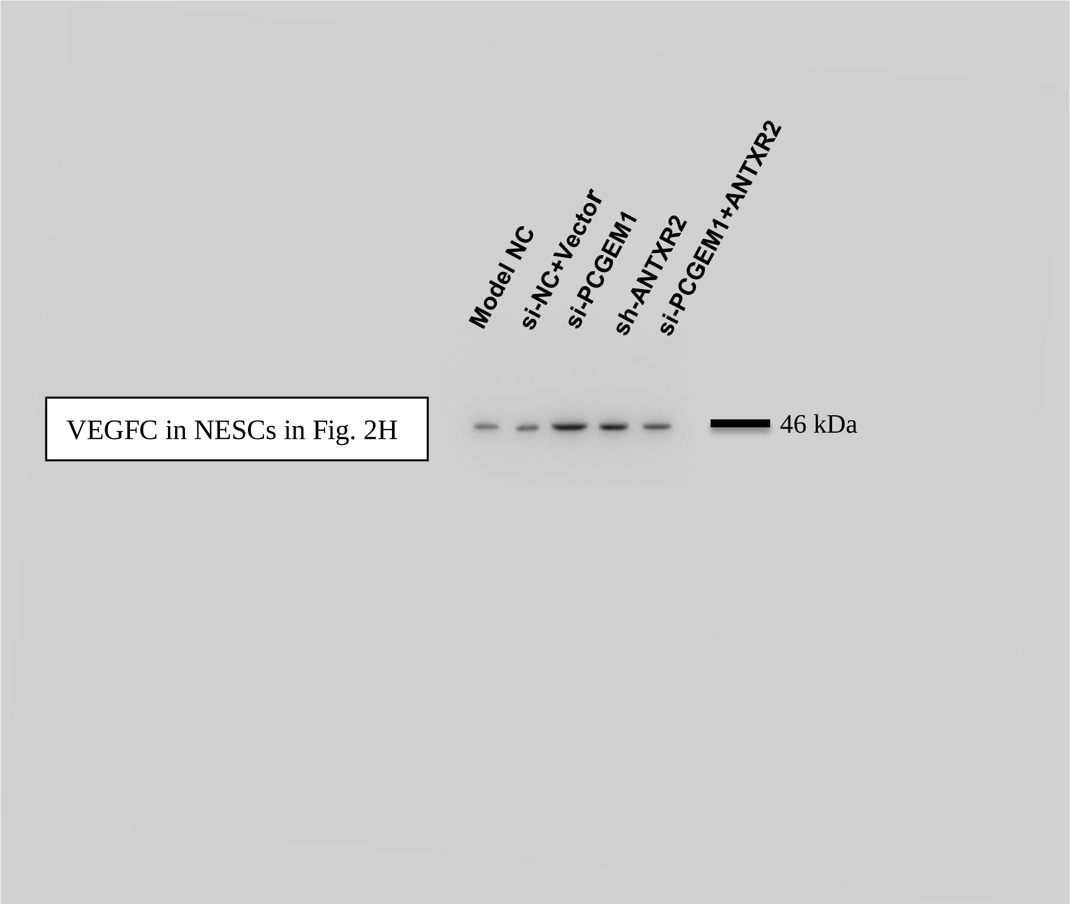
Model NC
si-NC+Vector
si-PCGEM1
sh-ANTXR2
si-PCGEM1+ANTXR2
VEGFC in NESCs in Fig. 2H
46 kDa

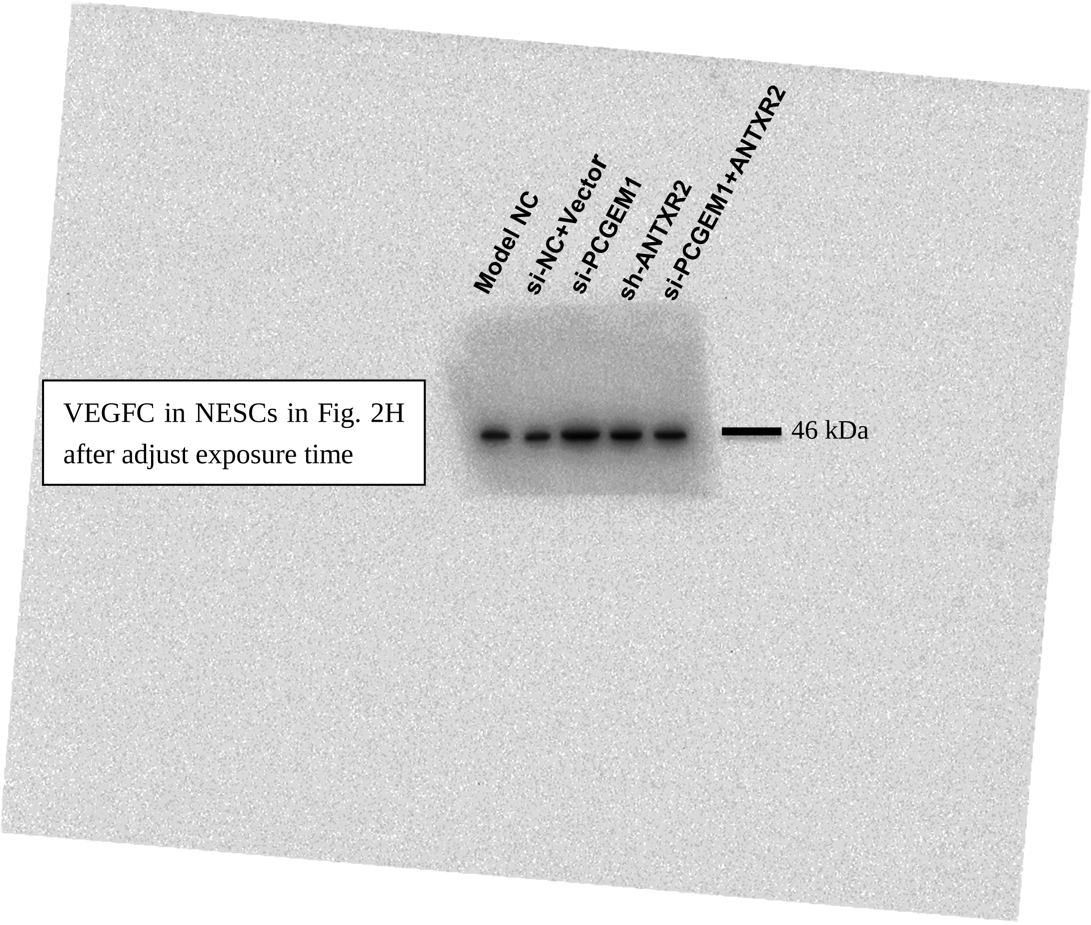
Model NC
si-NC+Vector
si-PCGEM1
sh-ANTXR2
si-PCGEM1+ANTXR2
VEGFC in NESCs in Fig. 2H
after adjust exposure time
46 kDa

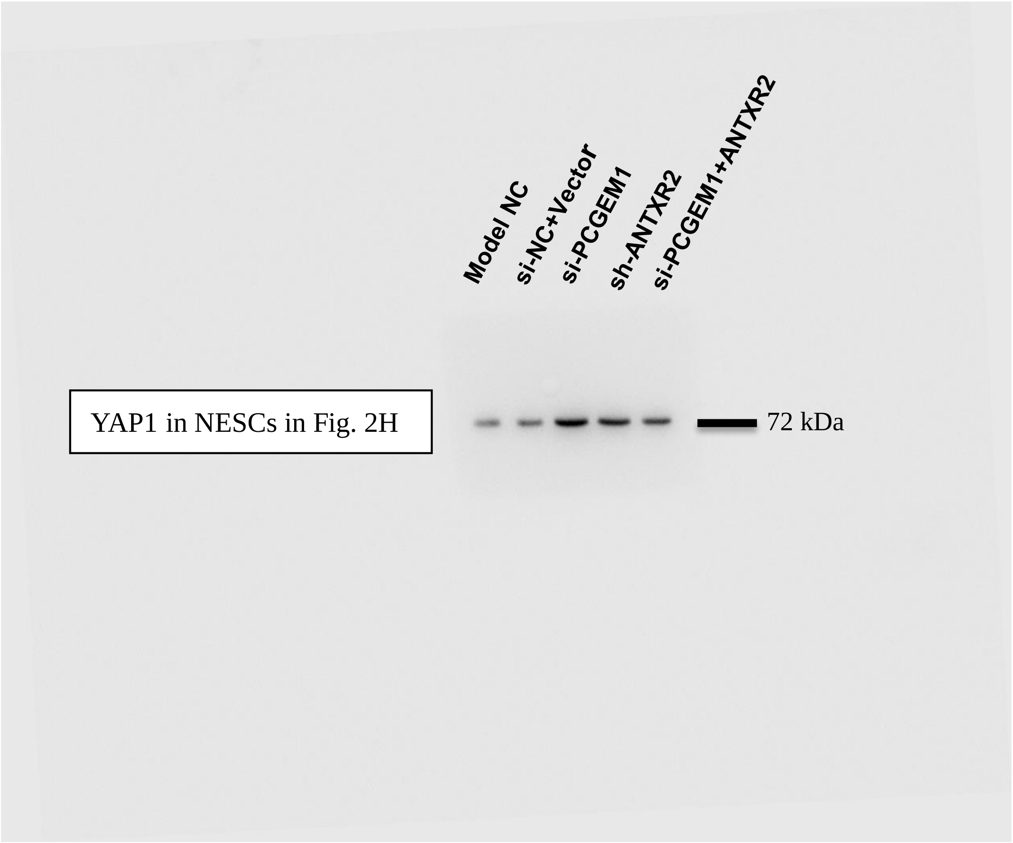
Model NC
si-NC+Vector
si-PCGEM1
sh-ANTXR2
si-PCGEM1+ANTXR2
YAP1 in NESCs in Fig. 2H
72 kDa

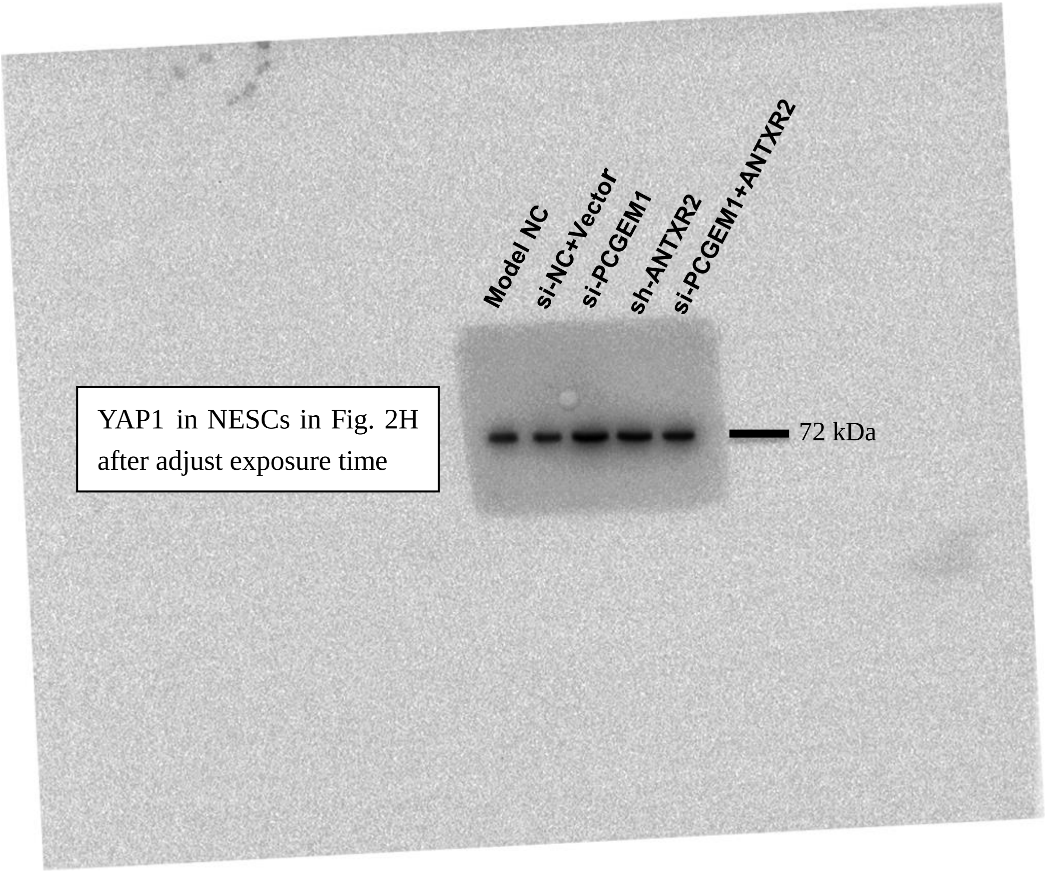
Model NC
si-NC+Vector
si-PCGEM1
sh-ANTXR2
si-PCGEM1+ANTXR2
YAP1 in NESCs in Fig. 2H
after adjust exposure time
72 kDa

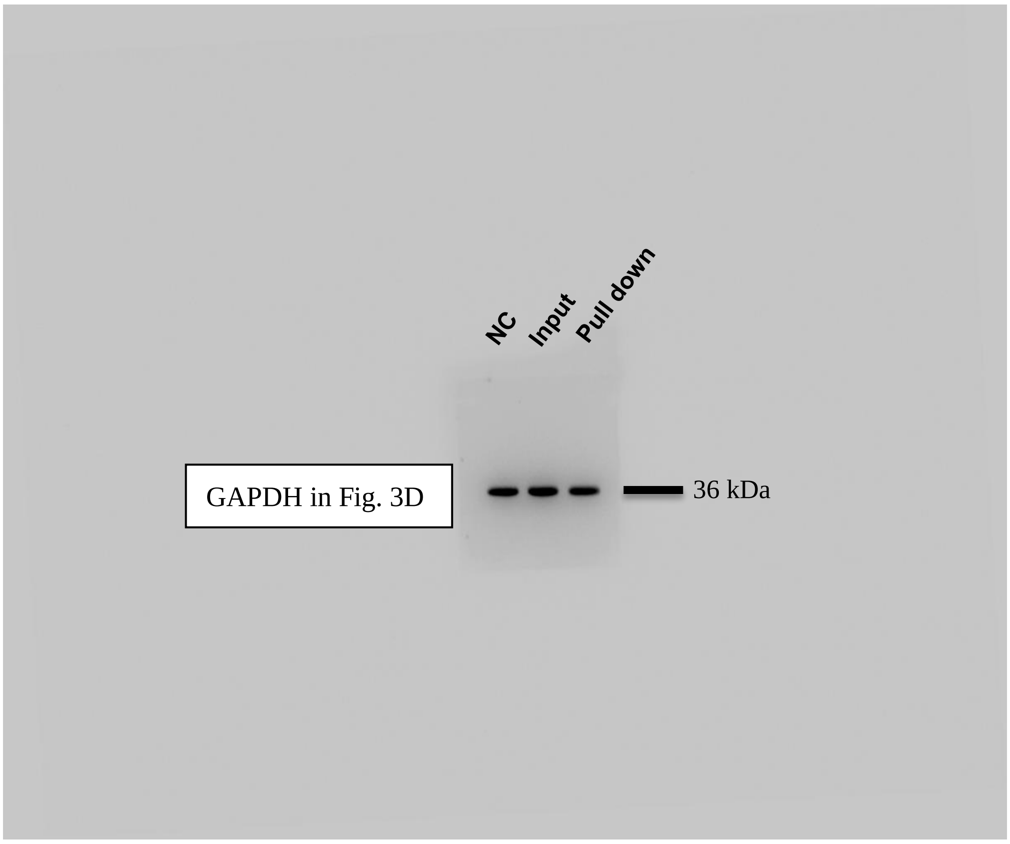
NC
Input
Pull down
GAPDH in Fig. 3D
36 kDa

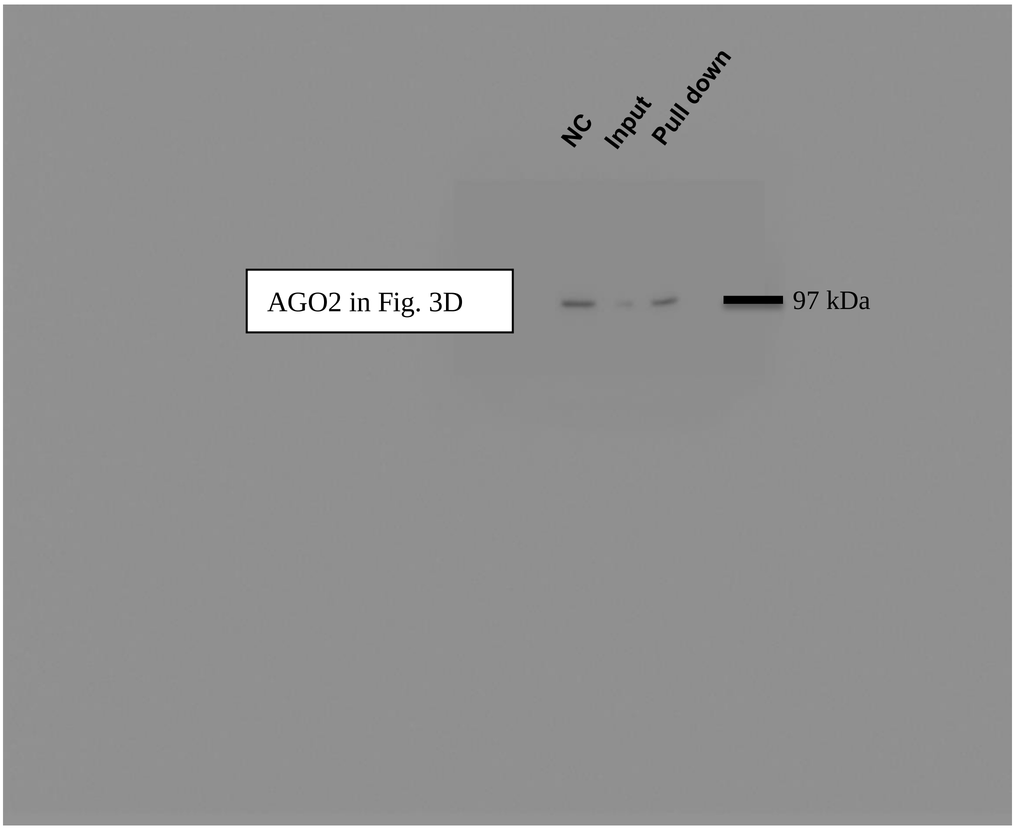
NC
Input
Pull down
AGO2 in Fig. 3D
97 kDa

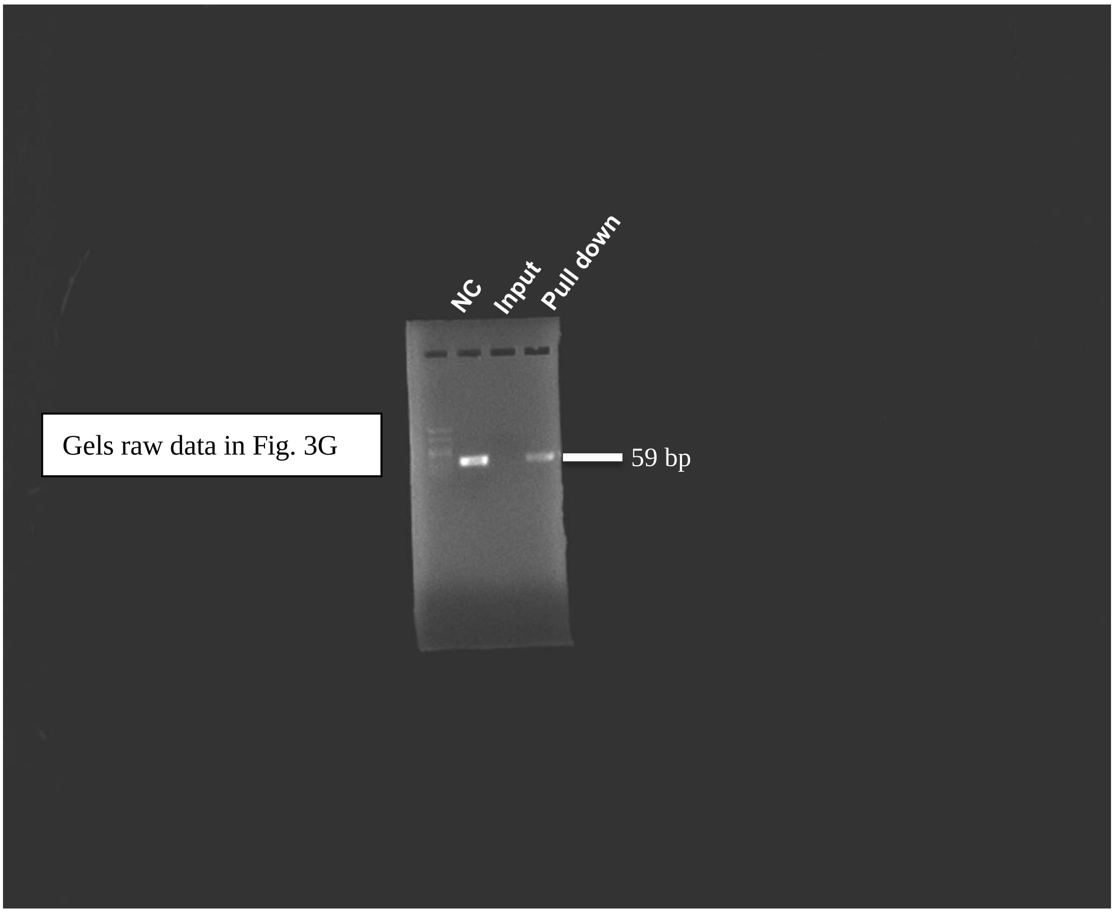
NC
Input
Pull down
Gels raw data in Fig. 3G
59 bp

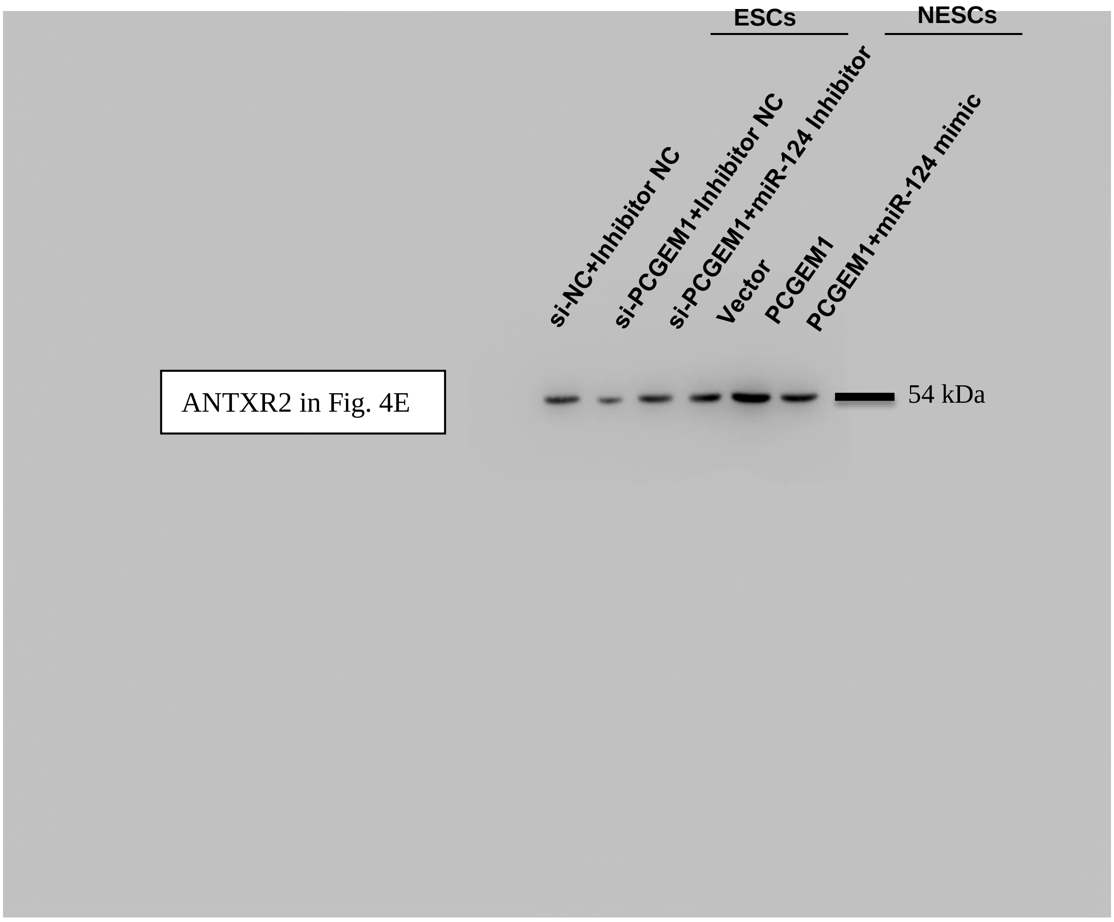
ESCs
NESCs
si-NC+Inhibitor NC
si-PCGEM1+Inhibitor NC
si-PCGEM1+miR-124 Inhibitor
Vector
PCGEM1
PCGEM1+miR-124 mimic
ANTXR2 in Fig. 4E
54 kDa

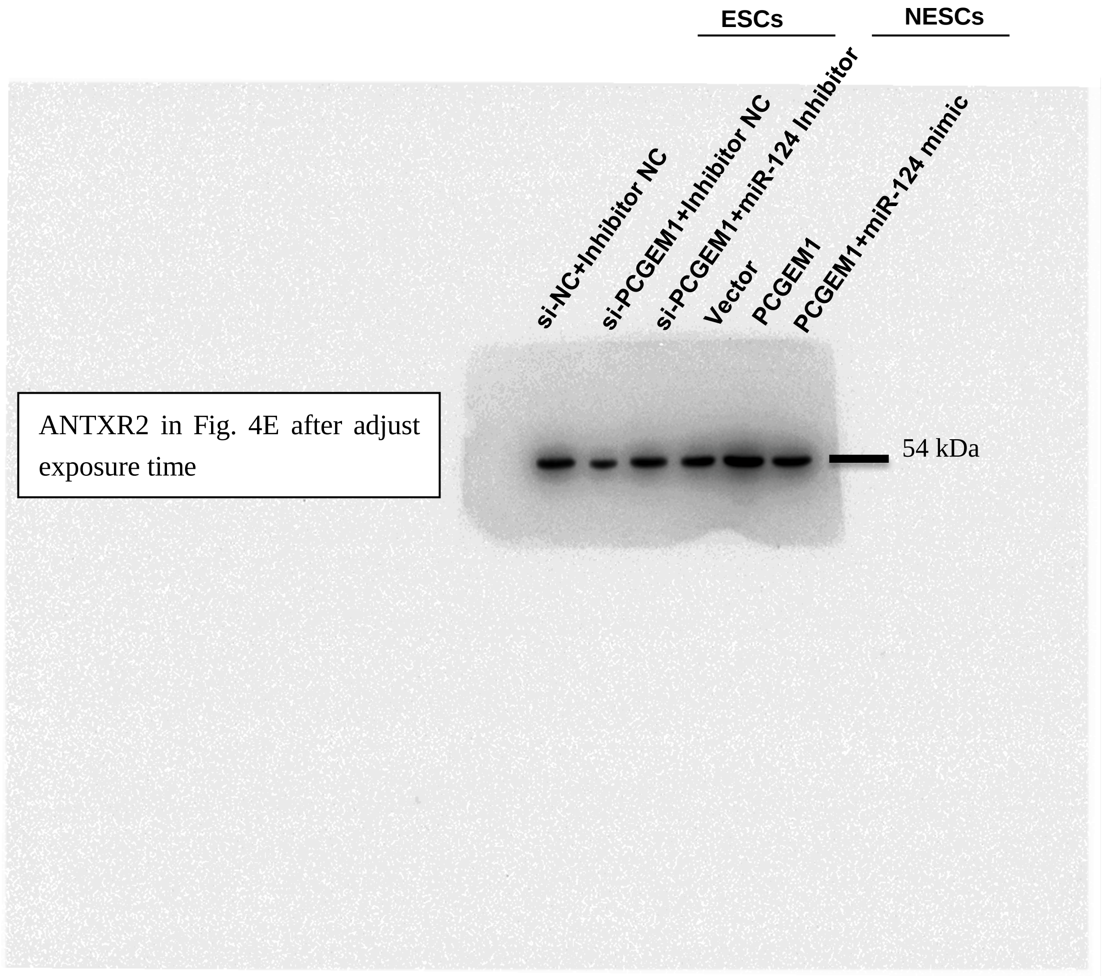
ESCs
NESCs
si-NC+Inhibitor NC
si-PCGEM1+Inhibitor NC
si-PCGEM1+miR-124 Inhibitor
Vector
PCGEM1
PCGEM1+miR-124 mimic
ANTXR2 in Fig. 4E after adjust exposure time
54 kDa

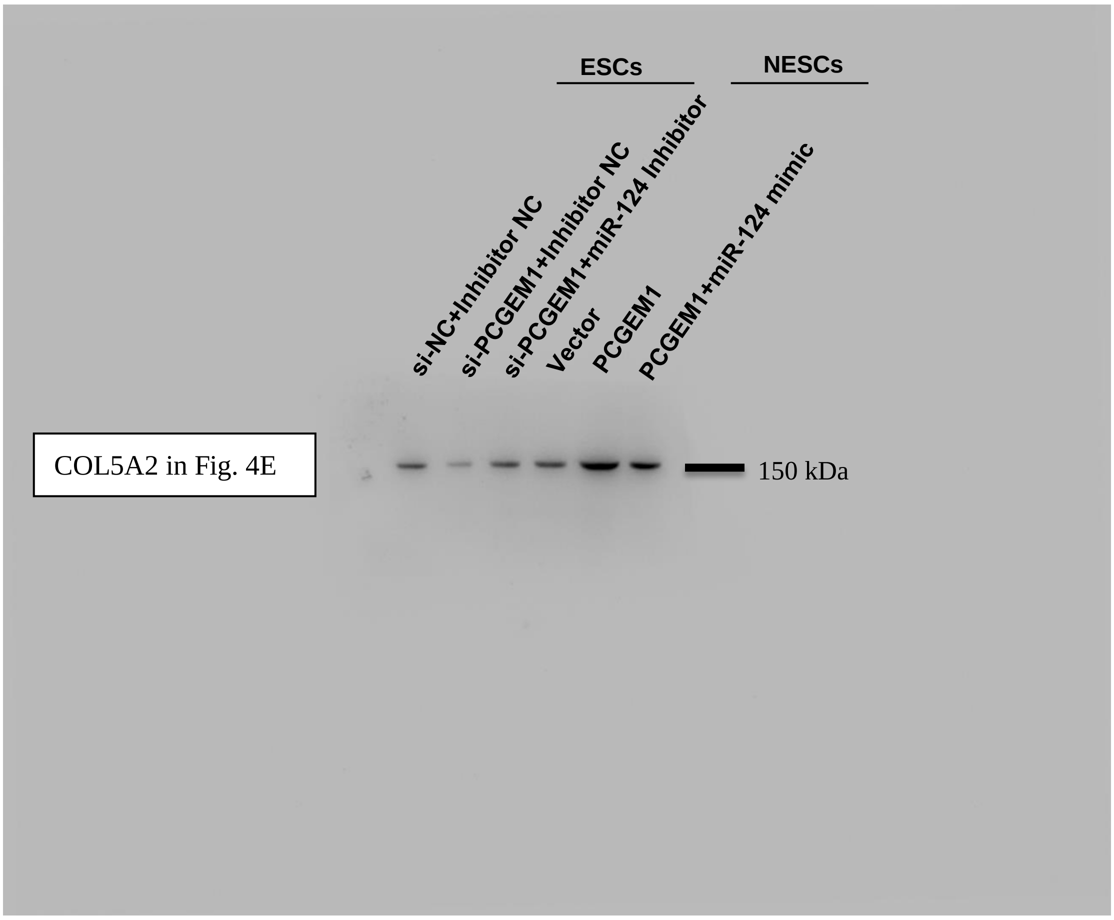
ESCs
NESCs
si-NC+Inhibitor NC
si-PCGEM1+Inhibitor NC
si-PCGEM1+miR-124 Inhibitor
Vector
PCGEM1
PCGEM1+miR-124 mimic
COL5A2 in Fig. 4E
150 kDa

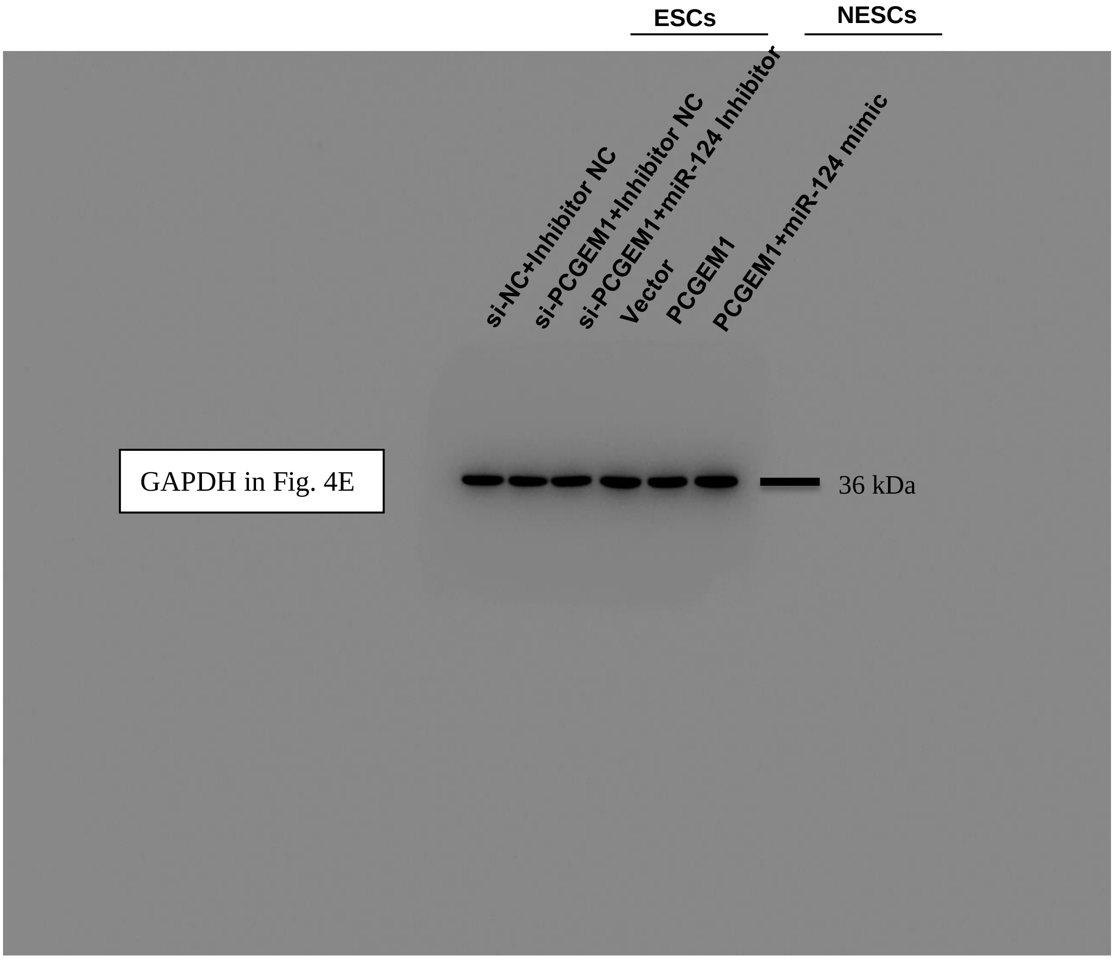
ESCs
NESCs
si-NC+Inhibitor NC
si-PCGEM1+Inhibitor NC
si-PCGEM1+miR-124 Inhibitor
Vector
PCGEM1
PCGEM1+miR-124 mimic
GAPDH in Fig. 4E
36 kDa

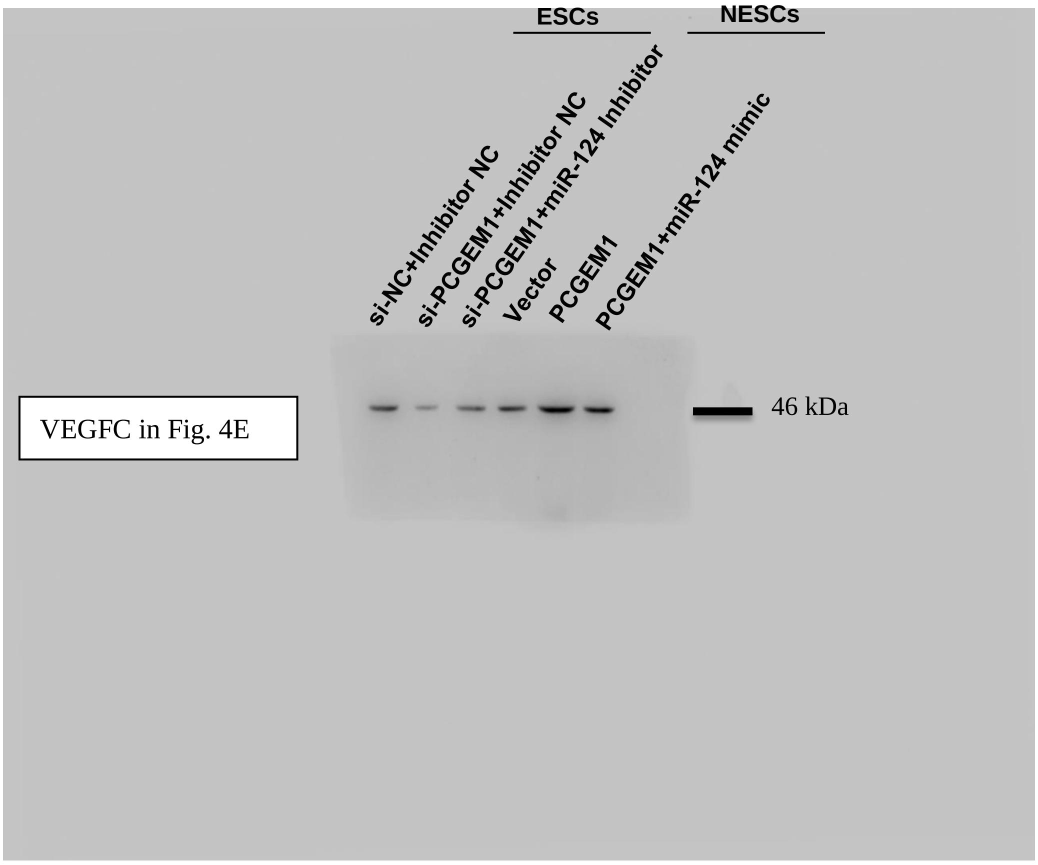
ESCs
NESCs
si-NC+Inhibitor NC
si-PCGEM1+Inhibitor NC
si-PCGEM1+miR-124 Inhibitor
Vector
PCGEM1
PCGEM1+miR-124 mimic
VEGFC in Fig. 4E
46 kDa

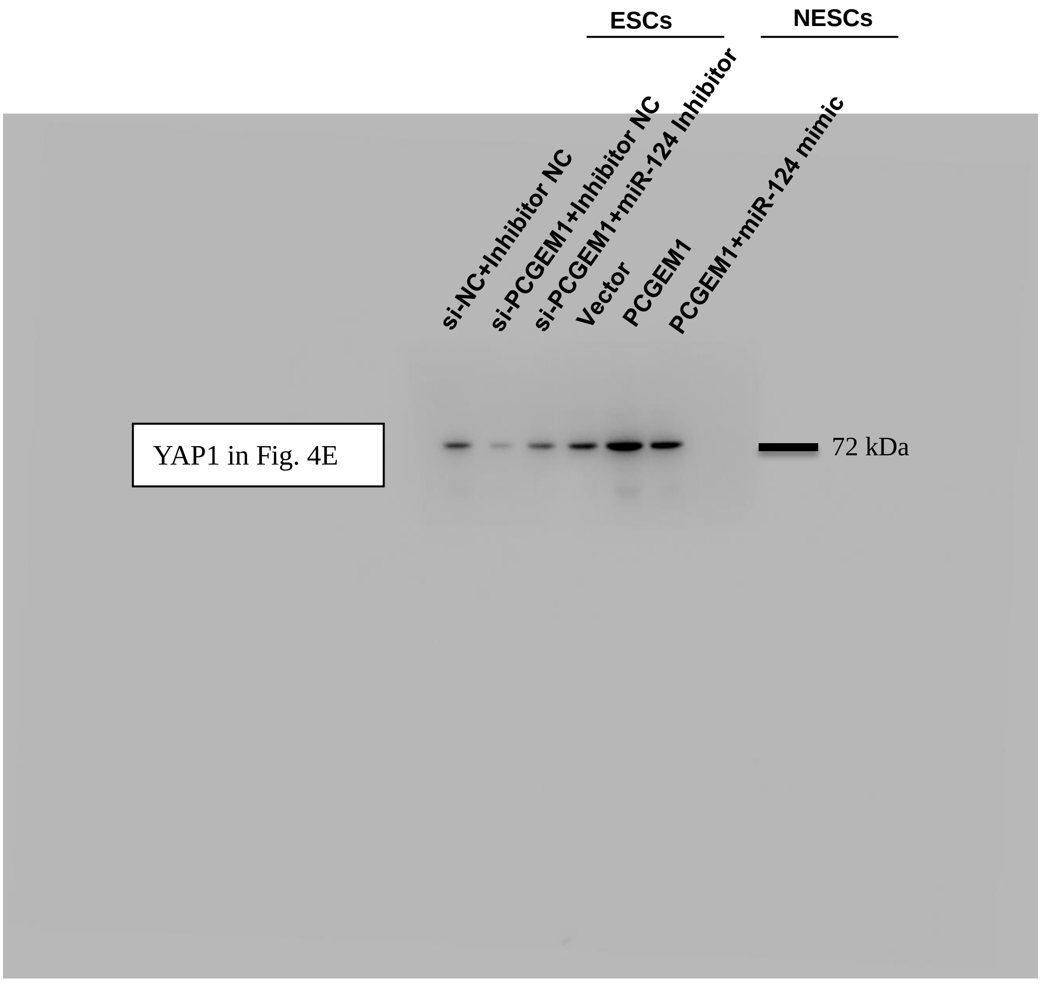
ESCs
NESCs
si-NC+Inhibitor NC
si-PCGEM1+Inhibitor NC
si-PCGEM1+miR-124 Inhibitor
Vector
PCGEM1
PCGEM1+miR-124 mimic
YAP1 in Fig. 4E
72 kDa
